# Spatial inference of *Culex pipiens* abundance and biting activity distribution in the Netherlands using citizen science

**DOI:** 10.1186/s13071-025-06774-3

**Published:** 2025-04-30

**Authors:** Ayat Abourashed, Catuxa Cerecedo-Iglesias, Martha Dellar, John R. B. Palmer, Frederic Bartumeus

**Affiliations:** 1 Viroscience, Erasmus University Medical Center, Rotterdam, Netherlands; 2 Centre d’Estudis Avançats de Blanes (CEAB-CSIC), Blanes, Spain; 3 Deltares, Utrecht, The Netherlands; 4 Department of Political and Social Sciences, Universitat Pompeu Fabra, Barcelona, Spain; 5 Centre de Recerca Ecològica i Aplicacions Forestals (CREAF), Cerdanyola del Vallès, Spain; 6 Institució Catalana de Recerca i Estudis Avançats (ICREA), Barcelona, Spain

**Keywords:** Citizen science, Mosquito surveillance, Species distribution model, *Culex pipiens*, Host–vector interaction, Mobile application

## Abstract

**Background:**

The expanding geographical spread of mosquito-borne diseases (MBDs) has intensified the need for effective mosquito surveillance. Additional surveillance, particularly of species such as *Culex pipiens*, is essential as this species is a key vector of West Nile and Usutu viruses. Citizen science offers an innovative approach to monitoring *Cx. pipiens* populations.

**Methods:**

Our study utilized data from the Mosquito Alert mobile app to model the spatial distribution and abundance of *Cx. pipiens* and mosquito bites during the summer of 2021 in the Netherlands. Using generalized linear mixed models, climatic and non-climatic factors were analyzed to create two distribution models of adult *Cx. pipiens* and mosquito bites as outcomes.

**Results:**

Population density, income, and agricultural areas (*P* ≤ 0.007) were identified as key determinants for both models. Blackbird population density, precipitation, and the interaction between artificial surfaces and temperature were also covariates for the *Culex* model, whereas sand and tree coverage were determinants for the bite model. The study controlled for biases in sampling effort to ensure robust predictions, revealing higher *Cx. pipiens* abundance in the central eastern areas of the country and widespread mosquito biting activity across the Netherlands.

**Conclusions:**

These findings underscore the importance of sociodemographic and environmental factors in mosquito distribution and biting dynamics, with citizen science emerging as a valuable tool for enhancing traditional surveillance. Future research integrating longer temporal datasets and human behavioral factors will further improve predictive accuracy and support more effective MBD prevention efforts.

**Graphical Abstract:**

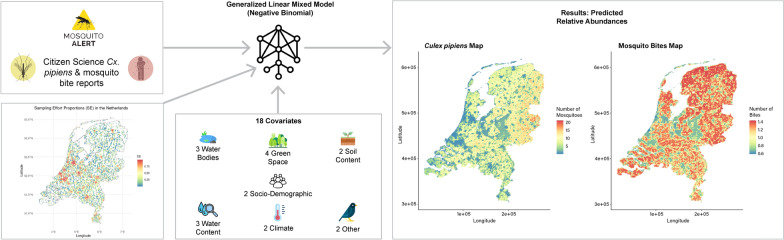

**Supplementary Information:**

The online version contains supplementary material available at 10.1186/s13071-025-06774-3.

## Background

The geographical spread of mosquitoes capable of transmitting disease pathogens is a growing global concern, leading to millions of human and animal deaths worldwide. This rise of mosquito-borne diseases (MBDs) underscores the growing importance of enhanced mosquito surveillance [1]. For instance, certain species, such as *Culex pipiens*, are known to be competent vectors of many MBDs, including West Nile virus (WNV) and Usutu virus (USUV) [2–4]. In recent years, European countries with low MBD prevalence (proportion of people in a population who have a disease at a given time), such as the Netherlands, have begun to experience autochthonous transmission of WNV and USUV, raising public health concerns [5–9]. In order to prevent further outbreaks or epidemics, innovative and enhanced routine vector surveillance of disease-competent vectors like *Cx. pipiens* is essential for implementing preventive intervention [10].

In the Netherlands, *Culex* mosquitoes are commonly found in urban and rural environments [11]. Their immature stages develop in stagnant water bodies such as ditches, ponds, and artificial containers. The abundance of these mosquitoes tends to peak during the warm summer months, influenced by both climatic and non-climatic factors such as temperature, precipitation, land use, and human population density [12–20]. *Culex pipiens* in particular has become quite abundant in more urban areas due to their ability to develop in a variety of locations [21, 22]. This, in combination with *Cx. pipiens* being a primary vector of WNV and USUV, poses public health issues with the increasing rates of urbanization, making the need for surveillance of this mosquito species urgent [23–25].

Targeted active surveillance of mosquitoes, such as using traps to collect adult individuals, is a common strategy to identify mosquito distribution and abundance. However, this strategy is expensive, time-consuming, and resource-dependent. Due to these restraints, the geographical scale of the surveillance system can be limited [26, 27]. Citizen science initiatives can effectively address limitations of traditional mosquito surveillance methods. This innovative approach facilitates more cost-effective mosquito surveillance while covering larger areas than more traditional professional systems [28, 29]. Moreover, citizen science not only can engage the public directly but can also enhance public health literacy [30–33]. Multiple citizen science mosquito surveillance projects across the world have already proved useful and reliable for monitoring mosquito distributions and nuisance [34–37].

Although citizen science does offer many benefits for expanding upon current surveillance, there are some limitations, such as sampling bias. For instance, Mosquito Alert (MA) is a mobile citizen science application that makes it possible for people to transmit reports about mosquito-related information [38]. Despite people having MA on their phones, however, their chances of submitting reports to MA will depend on situational factors such as what they are doing at the moment of observing a mosquito and where they are. Thus, the number of MA reports does not directly show how many mosquitoes are actually present in a given location. Nevertheless, this sampling bias can be mitigated by accounting for sampling effort, making it possible to improve estimates of actual mosquito population distributions in space and time [29].

Initially, MA focused on reporting adult invasive *Aedes* mosquitoes and their breeding sites, but in 2020, the mobile application was expanded to include mosquito bite reports and reports of other mosquito species, including those of the *Culex* genus. With reports submitted all across Europe, MA provides validated adult mosquito data at both the national and continental level, a first of its kind in mosquito citizen science. Each report is validated to determine mosquito species by three independent expert entomologists through manual inspection of digital images that participants transmit through the app. Each expert labels the report based on their confidence in identifying the target species in the photographs. If they are uncertain, they use a “not sure” label. Reports may be flagged if they require further review by a senior entomologist. The final taxonomic classification is determined by averaging the assessments of the three validators. Images from the Netherlands submitted to MA and validated as *Cx. pipiens* are likely to be grouped together with the two biotypes (*pipiens* and *molestus*) and the sibling species *Culex torrentium* [39–41]. Citizen scientists can also submit bite reports to MA; however, these reports cannot be validated yet as they do not include images [38, 42]. With these mosquito and bite reports, MA is able to collect valuable information about mosquito ecology, distribution, and nuisance at various spatial and temporal levels, with minimal costs and resources compared to traditional surveillance methods [38]. These reports have been indispensable in constructing predictive maps and even forecasting models for Barcelona [43]. Building such models is vital in understanding mosquito population distributions and mosquito biting dynamics to prevent and control potential MBD spread.

In 2020, the Netherlands experienced its first local transmission of WNV detected in mosquitoes, humans, and a bird [8, 9]. The emergence of these cases of MBDs in northern Europe raises concerns about the potential for wider disease spread. *Culex pipiens* is highly abundant in the Netherlands, and the country’s landscape provides suitable habitats for these mosquitoes to flourish [11, 44, 45]. The factors leading to these positive cases are still in question, but their occurrence shows the importance of regular mosquito monitoring and understanding of mosquito biting dynamics to prevent outbreaks. In 2021, MA was officially launched in the Netherlands, and thousands of reports were sent by citizen scientists in the country [46]. Using these reports, our study aims to create predictive, spatial models on the distribution and abundance of adult *Cx. pipiens* mosquitoes and mosquito bites in the Netherlands during the summer of 2021. Additionally, this study identifies climatic and non-climatic factors influencing mosquito activity.

## Methods

### Study area

The study area is the complete area of the Netherlands: latitude 50.75 N–53.55 N, longitude 3.35 W–7.22 E. The Netherlands is characterized by a mostly flat topography with diverse land cover that includes urban areas, agricultural lands, forests, wetlands, and extensive water systems. These landscapes play a significant role in mosquito distribution and abundance [47]. The country's intricate water network, including canals, rivers, and dikes, along with its temperate maritime climate characterized by moderate temperatures and high humidity, creates favorable conditions for mosquitoes [44, 47, 48]. During the study period (July 22, 2021, to August 22, 2021), the average temperature ranged from 12 °C to 15 °C, and the average daily rainfall was 3.025 cm.

### Data collection

#### Citizen science data

**Fig. 1:**
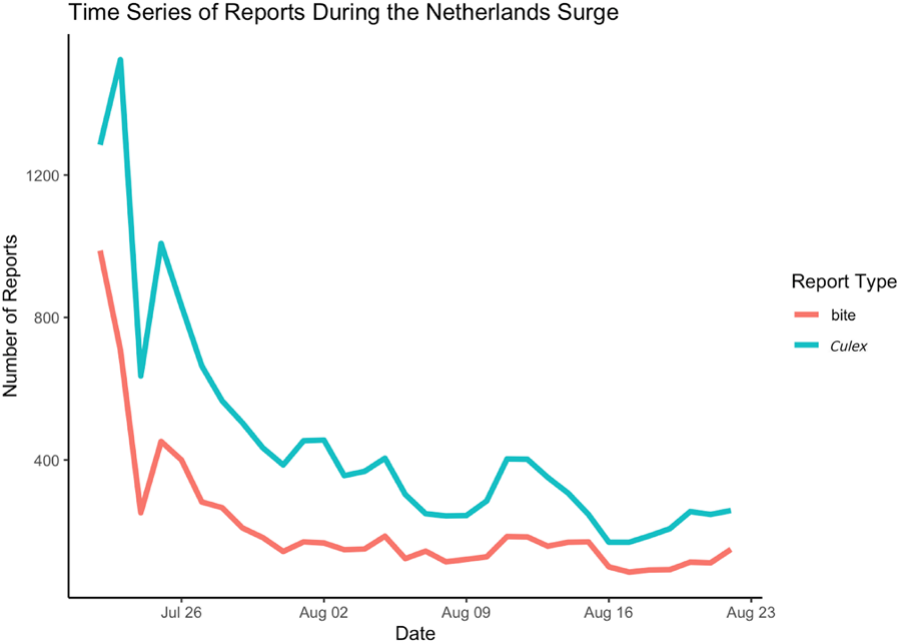
Time series of daily Mosquito Alert *Culex pipiens* (blue line) and bite (red line) reports in the Netherlands from July 22, 2021, to August 22, 2021

**Fig. 2:**
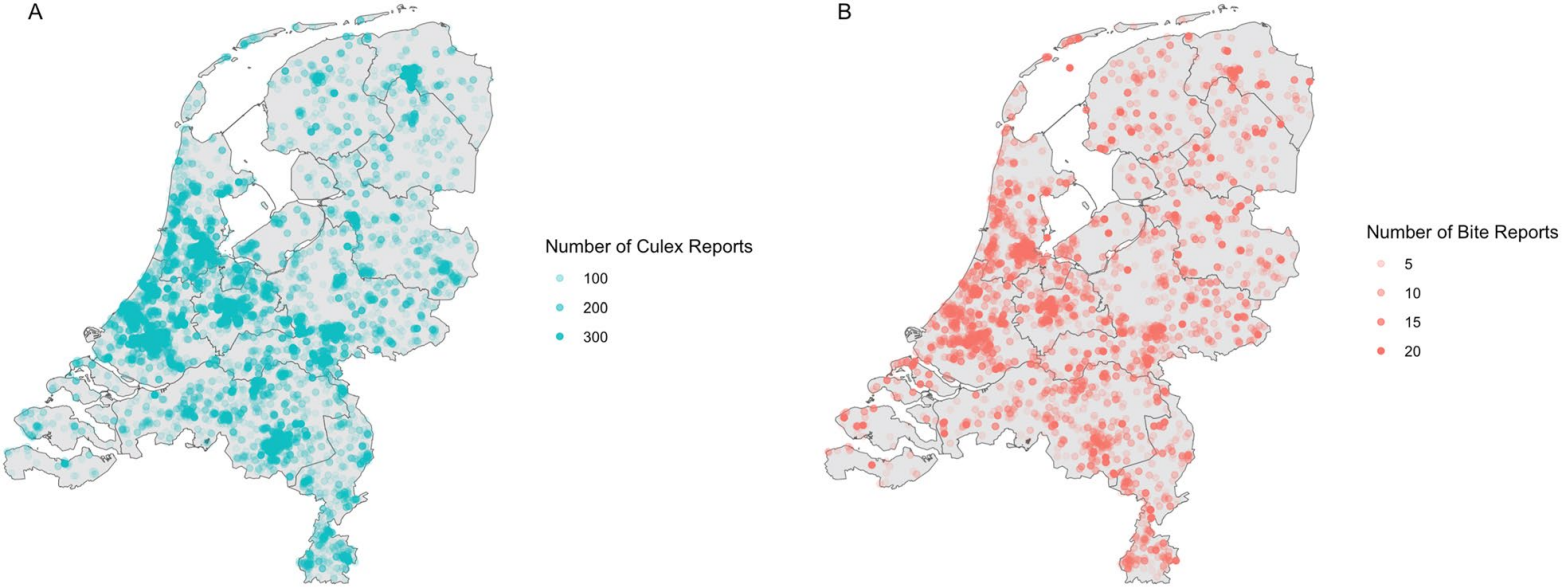
Daily totals of Mosquito Alert reporting across the Netherlands from July 22, 2021, to August 22, 2021, for **A** validated adult *Culex pipiens* reports and **B** bite reports

Data were collected from the MA citizen science project, focusing on adult *Cx. pipiens* reports and bite reports in the Netherlands. We rely only on the *Culex* reports that were classified by a team of expert entomologists as *Cx. pipiens* [38]. In this manuscript, we use the term “*Cx. pipiens*,” but it is possible that the reports are *Cx. pipiens/torrentium* complex and other *Culex* biotypes [39–41]. The mosquito bite reports are not classified by species, although some are linked to adult mosquito reports that are classified [38, 42]. On July 22, 2021, the MA application was officially launched to the Dutch public through Nederlandse Omroep Stichting (NOS), a national news channel [46]. This press release led to a large surge of MA reports from the Netherlands, resulting in a record number of submissions (14,405 *Cx. pipiens* reports and 6941 bite reports) within a 4-week time period from July 22, 2021, to August 22, 2021 (Figs. 1 and 2). All MA reports were filtered to include only those from the surge period in the Netherlands. Reports were aggregated by summing counts within 1 km^2^ cells.

#### Sampling effort data

**Fig. 3:**
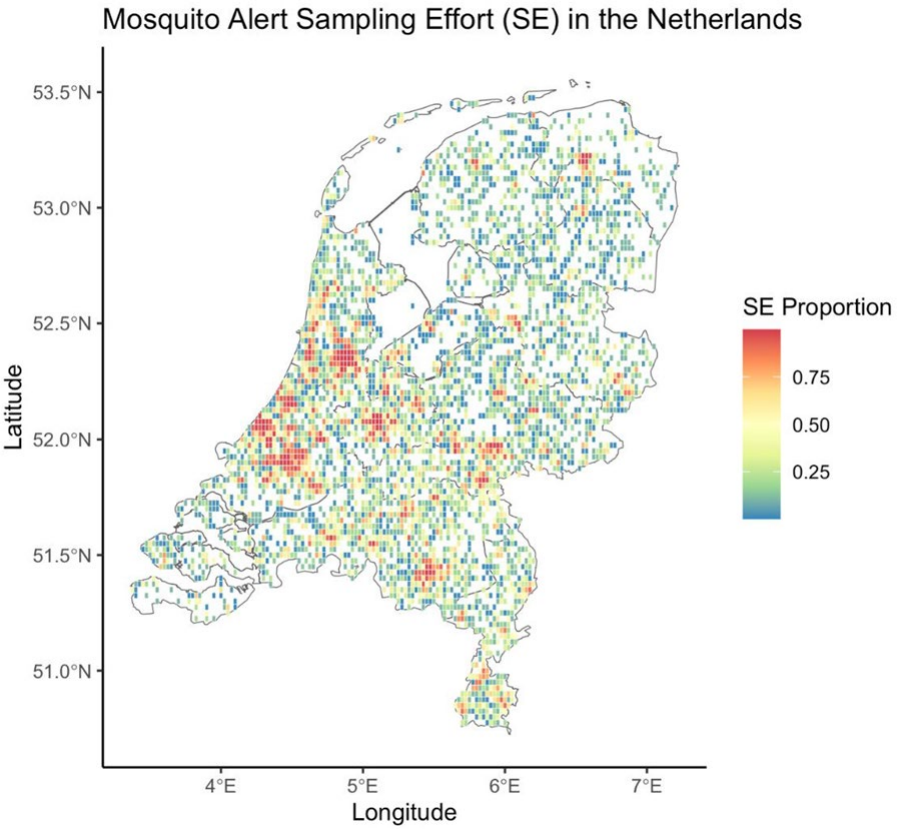
Mean sampling effort of Mosquito Alert citizen scientists by cell in the study period. SE, sampling effort

The sampling effort in each cell was determined following Palmer et al. [29] (Fig. 3). These authors calculated the probability of each active participant in a specific sampling cell submitting a report in the previous 2-week period based on the time elapsed since the participant downloaded the MA application [42]. This estimate is available at https://github.com/Mosquito-Alert/sampling_effort_data. For our analysis, we only considered the sampling effort during our study period. Then, we calculated a monthly estimate considering our own sampling cell by summing the individual probabilities for each cell during the study period. By accounting for sampling effort, we can fairly compare MA reports across areas with varying levels of data collection. Without this consideration, regions with more intense sampling might appear to have higher MA reports simply due to more data being collected, rather than a true difference in *Cx. pipiens* or bite occurrences.

#### Non-climate and climate covariates

Based on the literature, we identified 18 potential spatial covariates, two climatic and the rest non-climatic, that could be related to mosquito distribution and activity (Table 1) [13–15, 18, 20, 49–51]. All covariates covered the extent of the Netherlands, at 1 km resolution, and their values were standardized to ensure they were on a comparable range in the models.

**Table 1:** All variables included in analyses with their respective definitions and original resolution

Covariates	Definition	Resolution	References
Agricultural areas	Percentage of area used for horticulture under glass, grassland, gardening, arable land, or orchard	100 m	[52]
Clay	Percentage of clay in soil	500 m	[53]
Elevation	Meters above sea level (negative if below sea level)	25 m	[54]
Income	Average income per resident per municipality	Per municipality	[55]
msPAF	Multi-substance potentially affected fraction of aquatic species	1 km	[56]
Nitrogen	Percentage of nitrogen in soil	500 m	[53, 57, 58]
Artificial	Percentage of surface area with an artificial covering (e.g., concrete, asphalt)	100 m	[59]
Grass	Percentage of surface area which is grass	100 m	[60]
Permanent wet	Percentage of area which is wet (not water) 75% of the time, e.g., reeds, peat land, inland wetlands, and coastal wetlands (incl. salt marshes)	100 m	[61]
Population	Human population density	1 km	[62]
Salinity	Depth of freshwater/saltwater boundary	250 m	[63]
Sand	Percentage of sand in soil	500 m	[53, 64, 65]
Temporary water	Percentage of area which is water 25–85% of the time, e.g., temporary water surfaces associated with permanent water bodies, temporary natural (e.g. steppe) lakes and temporary artificial lakes (e.g., cassettes of fish ponds), intermittent rivers, flood areas, water-logged areas, wet agricultural fields, including rice fields, intertidal areas	100 m	[66]
Temporary wet	Percentage of area which is wet (not water) 25–75% of the time, e.g., areas of changing soil moisture, inland saline marshes, intermittent wetlands	100 m	[66]
Tree coverage	Density of tree coverage	100 m	[67]
Blackbirds	Density of *Turdus merula* (blackbird) population	1 km	[68]
Precipitation	Mean precipitation during study time period	1 km	[69]
Temperature	Mean temperature during study time period	1 km	[70]

### Species distribution modeling based on non-biased citizen reporting activity

To understand the spatial patterns of *Cx. pipiens* and bites, we fitted generalized linear mixed models (GLMMs). The response variables were (1) the number of adult *Cx. pipiens* reports and (2) the number of bite reports per cell. We first examined outliers in the response variables and removed values that fell beyond the 90th and 85th percentiles of the distributions for *Cx. pipiens* and biting reports, respectively (see Additional file 1: Fig. S1). Secondly, distributions of both variables (*Cx. pipiens* report counts and biting report counts) were visually assessed using boxplots and histograms. We selected the best-fit model among three different distributions (negative binomial, Poisson, and geometric). For both cases, we selected the negative binomial distribution with a log link function to describe the relationship between the response and the predictor variables (see Additional file 1: Fig. S2). The negative binomial distribution is commonly used in species distribution models based on count data, particularly when there is overdispersion [71]. The sampling effort was incorporated into the models as an offset. Since the models correct for sampling bias, the reports are used as fair estimates of mosquito abundance (*Cx. pipiens* image reports) and activity (biting reports). To address region-specific variation, random effects were added to the model using the second-level administrative divisions (municipalities) in the Netherlands. In addition, we tested for interactions among environmental covariates to capture complex relationships.

Final models for *Cx. pipiens* abundance and the bite abundance model were obtained using a stepwise backward procedure based on the Akaike information criterion (AIC) and Bayesian information criterion (BIC), with a threshold of 2 [72]. Additionally, we checked for multicollinearity (i.e., excessive correlation among covariates) using the variance inflation factor (VIF), removing covariates with VIF values over 3 to avoid collinearity [71]. The goodness of fit was also assessed by means of calculating the pseudo-R squared (pseudo-R^2^). Then, model cross-validation was performed through a cross-validation set approach. We randomly divided the dataset into training (80%) and testing (20%) sets. The model was trained again on the training dataset, and the resulting model was then used to calculate predictions for the testing dataset. We also calculated a range of evaluation statistics to assess the predictive performance using Pearson’s correlation coefficient (*r*) and Spearman’s rank correlation (*S*).

Finally, prediction maps were generated by applying the final models to the entire study area considering only the study period. The maps display the predicted abundance of *Cx. pipiens* and bite activity across the study region.

All analyses were conducted in R version 4.4.1 [73]. Packages used for analysis included *MASS*, *glmmTMB*, *performance*, and *DHARMa*.

## Results

### Determinants of *Cx. pipiens* and bite activity

#### *Cx. pipiens* model

Variables selected for the final *Cx. pipiens* count prediction are shown in Table 2. Agricultural areas, human population density (Population), Precipitation, and the interaction between temperature and surfaces with artificial coverings (Artificial) had a positive relationship with *Cx. pipiens* abundance. However, Income and the population density of blackbirds (Blackbirds) were negatively related to *Cx. pipiens* abundance. All variables were statistically significant. The pseudo-R^2^ was 0.487. All VIF values were below 2, indicating low multicollinearity among these variables.

**Table 2:** Generalized linear mixed model results for Mosquito Alert *Culex pipiens* reports

Coefficients	Estimate	SE	*P*-value	VIF
(Intercept)	1.482	0.033	< 0.001	–
Agricultural areas	0.193	0.018	< 0.001	1.324
Income	−0.104	0.026	< 0.001	1.006
Population	0.144	0.017	< 0.001	1.421
Blackbirds	−0.033	0.014	0.020	1.130
Precipitation	0.070	0.021	0.001	1.043
Artificial × Temperature	0.085	0.014	< 0.001	1.023

*SE* standard error, *VIF* variance inflation factor

The evaluation statistics from the cross-validation set approach showed a robust correlation between the observed and predicted values. Specifically, Pearson’s correlation was reported at *r* = 0.71, while Spearman’s rank reached *S* = 0.7. This strong correlation underlines the robustness of the predictive model, confirming its effectiveness in capturing the underlying patterns in the data (see Additional file 1: Fig. S4).

QQ and residual plots to validate the models are included in the supplemental material (Additional file 1: Fig. S3).

#### Bite model

Table 3 shows the variables included in the final bite count predictions, which were all statistically significant. Agricultural areas, human population density (Population), and Tree Coverage had a positive relationship with bite count. Income and Sand were negatively related to bite numbers. The goodness of fit for the model (pseudo-R^2^) was 0.465. The VIF values suggest that there is no multicollinearity concern, since all values are less than 2, indicating low correlation between the variables.

**Table 3:** Generalized linear mixed model results for Mosquito Alert bite abundance

Coefficients	Estimate	SE	*P*-value	VIF
(Intercept)	1.828	0.025	< 0.001	–
Agricultural areas	0.081	0.014	< 0.001	1.394
Income	−0.053	0.020	0.007	1.005
Population	0.081	0.013	< 0.001	1.493
Sand	−0.062	0.018	< 0.001	1.179
Tree coverage	0.061	0.013	< 0.001	1.242

*SE* standard error, *VIF* variance inflation factor

In this case, the predictions also closely mirrored the observations, as illustrated in the evaluation statistics from the cross-validation set approach (*P* = 0.77 and *S* = 0.76; see Additional file 1: Fig. S8).

QQ and residual plots to validate the bite models are included in the supplemental material (Additional file 1: Fig. S7).

### Spatial distribution of *Cx. pipiens* and bite activity

**Fig. 4:**
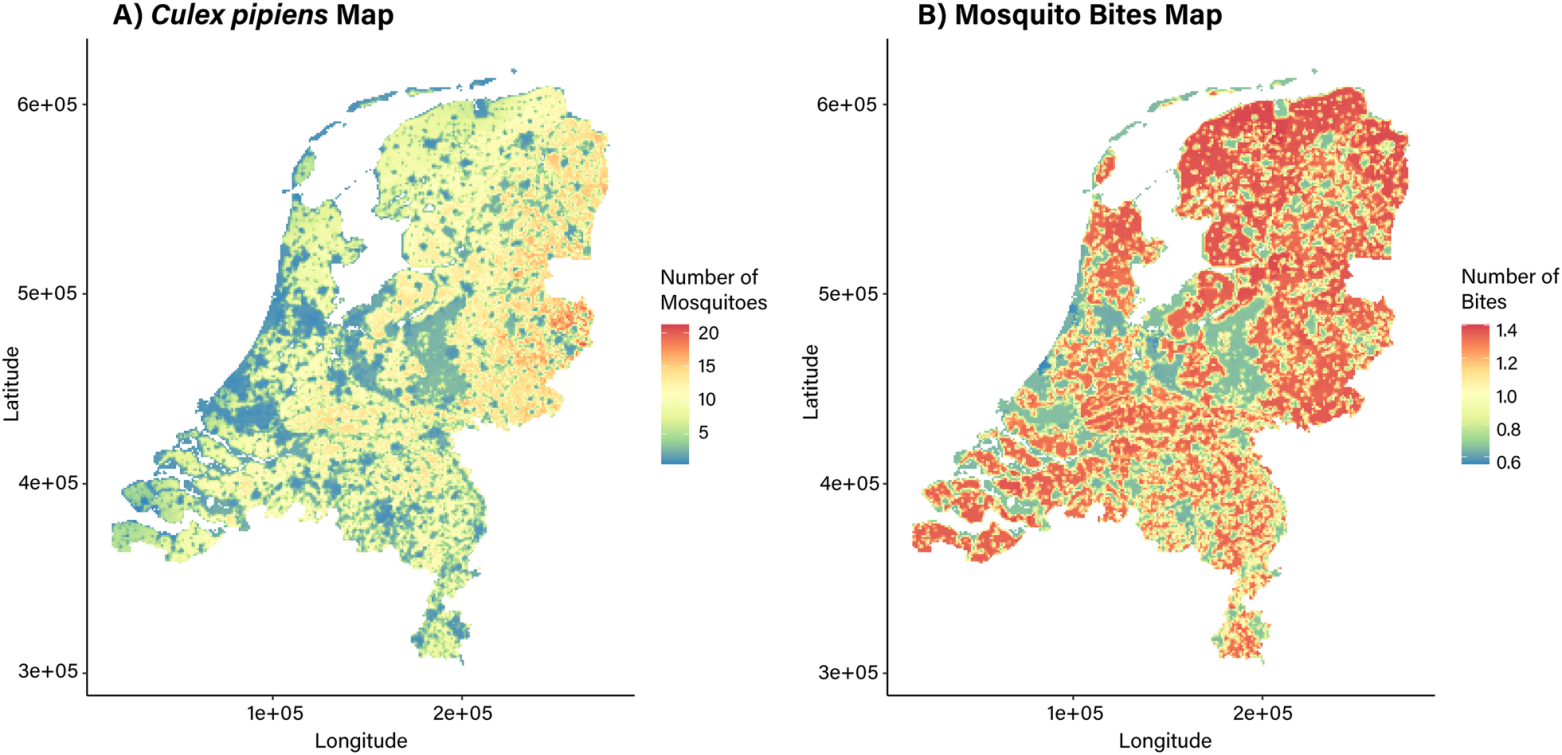
Spatial prediction maps based on Mosquito Alert citizen science data controlling for sampling bias: **A** *Cx. pipiens* abundance and **B** bite abundance across the Netherlands

Spatial predictions of the two models show some differences (Fig. 4). The predicted *Cx. pipiens* map (Fig. 4A) indicates most areas in the Netherlands with a moderate predicted relative abundance of *Cx. pipiens* mosquito reports (yellow), suggesting that *Cx. pipiens* mosquitoes are abundant throughout the country. However, there is some variation in the relative abundance. The highest predicted abundance appears concentrated in the northeastern and central eastern regions of the Netherlands (orange and red). There also seems to be some high areas of *Cx. pipiens* abundance in the middle of the country, in areas with more green areas. Areas along the western coastline, the northern islands, and national parks show very low abundance of *Cx. pipiens*.

To contrast, bites seem to be highly abundant and show a wide spread throughout the Netherlands (Fig. 4B). Both dense, urban areas and national parks seem to have low bite occurrence, whereas residential and rural areas have more bites. Areas with water have low—near zero—probability for occurrence of mosquito bites.

### Relationship between predicted abundance of *Cx. pipiens* and biting activity

**Fig. 5:**
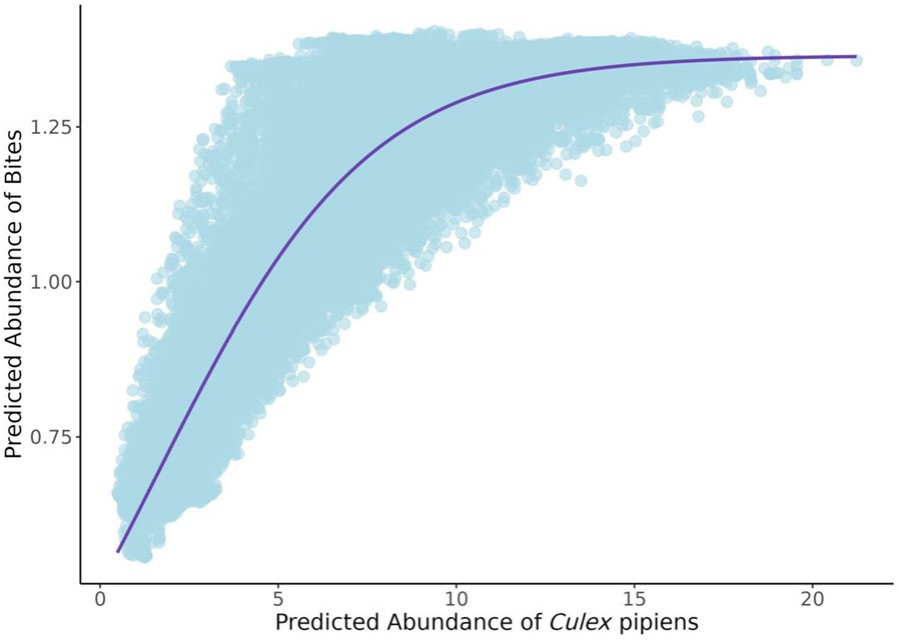
Scatter plot of the predicted abundance of *Cx. pipiens* and bites

Figure 5 shows the relationship between the predicted abundance of *Cx. pipiens* (*x*-axis) and the predicted abundance of bites (*y*-axis). The scatter plot reveals a non-linear trend where predicted bites increase rapidly at lower mosquito abundance but plateau at higher *Cx. pipiens* abundance. A fitted curve highlights this saturation effect, suggesting that beyond a certain mosquito abundance, additional mosquitoes do not proportionally increase the number of bites reported.

## Discussion

After accounting for biases caused by opportunistic reporting (i.e., convenience sampling), the models predicting the number of reports submitted by citizen scientists demonstrated reasonable predictive power. However, there is still room for improvement. We suggest that these types of models could help infer relative mosquito abundance and biting intensity. Through citizen science reports, the MA initiative in the Netherlands can provide valuable insights into the spatial distribution patterns of mosquitoes and their nuisance activity. Although this study focuses on a specific window of mosquito seasonality in the Netherlands and is thus a bit limited and imperfect, it provides further understanding of real-time human–mosquito interactions and mosquito biting activity across the whole country.

While there were some differences in key covariates between the *Culex* model and the bite models, Population and Income were significant sociodemographic covariates for both models. By including the sampling effort as an offset, the models control, to some extent, that the observed association between Population and the response variables is not merely due to higher data collection in populous areas but reflects a genuine increase in the abundance of *Cx. pipiens* mosquitoes and mosquito biting activity. Hence, the positive and significant relationship of Population suggests that human density plays a role in the interaction and attraction between mosquitoes and humans [74–77]. More populated areas often provide new breeding site opportunities for certain *Culex* mosquitoes (e.g., the biotype *molestus*), as well as more human hosts for certain *Culex* mosquitoes to bite in general [21, 45, 76, 78]. Interestingly, Income was negatively related to both *Culex* and bite abundance. Not surprisingly, socioeconomic factors do play a role in mosquito abundance and activity. A study in the United States found that *Cx. pipiens* pupae were more likely to be found in neighborhoods with lower median incomes in Baltimore, Maryland, and Washington, DC [79]. This suggests that lower-income residents may have more exposure to disease vectors. However, a previous comparative study showed the opposite in other *Culex* species, contrasting with our results [80]. Overall, these findings highlight the interplay between sociodemographic factors and *Cx. pipiens* and biting patterns, underscoring the need for targeted public health interventions, especially in vulnerable communities.

For both the *Cx. pipiens* and bite count models, Agricultural areas was a significant land coverage predictor. *Culex pipiens* tend to breed in standing water, which is common in agricultural settings. Agricultural landscapes provide various types of standing water habitats, such as irrigation ditches, drainage channels, livestock troughs, and nutrient-rich water bodies, which are ideal breeding sites for *Cx. pipiens* larvae [81, 82]. Although *Cx. pipiens* is typically more abundant in urban and peri-urban areas, this mosquito is able to adapt to different habitats, making it highly abundant across the Netherlands [11, 21, 22]. It could also be that the expert validations erroneously classified *Cx. torrentium* images as *Cx. pipiens*, given that the two species are difficult to distinguish without dissecting physical specimens. *Culex torrentium* might be a species to consider relevant in agricultural areas [83]. Further complicating species identification, the females of the sensu stricto biotypes and *torrentium* cannot be distinguished by classic morphology, although the taxa may differ ecologically [84]. A recent study analyzed the feeding habits of these subtypes in the Netherlands, finding differences influenced by host availability. In urban environments, they observed more frequent human blood-feeding in residential areas compared to park areas [85]. Another comprehensive study reported mixed blood meals across the group, combining avian, human, and non-human mammalian sources [86]. This finding is particularly noteworthy as it highlights the potential transmission risk between different host species. These results emphasize the importance of considering diverse habitat types when assessing mosquito breeding patterns and developing effective vector control strategies.

Interestingly, only two of the climatic variables considered (Precipitation and Temperature) were selected for the *Cx. pipiens* model. This may be due to the limited data collection timeframe, which captured only a “snapshot” of mosquito distribution during suitable conditions. Precipitation varied substantially across the country even within this snapshot, so it is not surprising that it was an important variable in the models. After rainfall, various surfaces may fill with water and enable new breeding site proliferation, providing suitable habitats for different life stages of the mosquito [15]. Additionally, in more urban areas, rainwater can stay on impermeable surfaces, offering other potential habitats for mosquitoes to breed [87]. While Temperature showed less spatial variation, its interaction with artificial surfaces (Artificial) was a positive predictor for *Cx. pipiens* abundance. Artificial surfaces tend to be in urban environments; therefore, this interaction with warm weather suggests that urban heat islands (UHIs) could exacerbate mosquito populations, a phenomenon observed in other studies [88–91]. This is a cause for concern, as the increase in UHIs, as well as higher temperatures across the country, can cause the spread of disease vectors and accelerate virus replication times, enabling disease transmission in places that were previously considered unsuitable. Again, these revelations underscore the importance of proactive vector control management, especially in growing urban areas.

In the *Cx. pipiens* model, Blackbird population density emerged as a significant negative predictor for mosquito count. While a previous study found blackbirds to be abundant in the eastern part of the Netherlands, they had an even higher abundance in urban areas [68]. Our findings suggest that *Cx. pipiens* are less prevalent in urban environments, which could potentially account for the observed negative relationship. However, further research is needed to explore this dynamic, particularly given the role of blackbirds as known transmitters of WNV [2, 23, 68].

The predictive spatial maps for *Cx. pipiens* and bite abundance had some similarities and differences. The *Cx. pipiens* spatial map indicates fairly widespread, heterogeneous distributions of *Cx. pipiens* across the Netherlands. Areas with the highest *Cx. pipiens* estimates are in the center of the country near the eastern border, near Germany. This area is in the municipality of Twente (Overijssel province), which is mostly rural [92]. With a homogeneous spread of biting activity, there is a high probability that people will be bitten at least once, especially given the high counts of *Cx. pipiens* mosquitoes throughout the country. Further studies are necessary to reveal these differences in *Cx. pipiens* abundance. Understanding these spatial patterns is crucial for public health planning, as it helps identify high-risk areas for potential MBD transmission. While the Netherlands is not considered as having a prevalence for MBDs, recent cases of WNV and Usutu are cause for concern [6, 8, 9]. Over time, these maps could be quickly generated in crisis scenarios (e.g., after a major flooding event) and guide vector control interventions, such as ultralow-volume sprays [27, 36, 49]. If citizen scientists submit reports in a more routine manner during mosquito season, these maps can also aid in vector surveillance efforts (e.g., where to allocate traps) [28, 29, 34, 35]. By pinpointing regions with elevated mosquito and biting activity, targeted vector control and community education efforts can be implemented to reduce disease risk and protect public health.

Other studies have made similar *Cx. pipiens* abundance maps for the Netherlands based on data from traditional trapping methods [93, 94]. They also found low abundance in national parks and higher abundance in rural areas and also found lower abundance in urban areas [93]. In the future, the integration of traditional surveillance alongside citizen science campaigns can provide an additional way to validate these results and to improve vector activity and abundance predictions at the country level. As for now, it is highly encouraging that citizen science methods can reproduce similar spatial patterns to these more traditional studies. It suggests that citizen science could be a valuable tool moving forward, enabling researchers and competent authorities to combine traditional surveillance data with novel data streams coming from participatory science, to improve knowledge and predictions, and produce more cost-effective interventions [35].

Interestingly, the relationship exemplified between predicted *Cx. pipiens* abundance and bite abundance likely reflects biological constraints on mosquito biting behavior, particularly due to blood digestion times and the limits imposed by the gonotrophic cycle [21]. Once a mosquito takes a blood meal, it must digest it before seeking another host, temporarily removing it from the active biting population [21, 83]. At higher mosquito density, a significant proportion of the population is likely in this post-feeding, non-biting phase, causing a natural ceiling in observed bite reports [18, 19]. Additionally, host avoidance behavior and mosquito dispersal could further contribute to the observed saturation, suggesting that simply an increasing mosquito presence does not lead to a linear increase in biting pressure [16, 17]. These findings have implications for vector surveillance and control, indicating that bite-based monitoring may underestimate true mosquito abundance at high population levels.

This study does have some limitations. Since the majority of MA reports from the Netherlands are from a very specific period, we created spatial models, with no temporal component. Ideally, we would have MA data that spanned the entirety of the mosquito season and over the course of multiple years, leading to more robust prediction models. Other climate variables such as relative humidity and wind speed have an effect on *Culex* populations and biting behavior throughout the season [3, 45, 49, 95, 96], so it could also be useful to include these. Considering that this is citizen scientist-submitted data, there is a human behavioral component that is not accounted for in our models, which might affect estimates. For example, people may use preventive measures to avoid being bitten by mosquitoes. In the Netherlands, over 90% of participants in a recent survey reported using at least using one prevention measure, with preference for skin repellents and plug-in repellents [97]. Although the communication campaign in the Netherlands was focused on WNV vectors like *Cx. pipiens*, and all of the submitted adult mosquito images were of this species, the bite reports could not be validated at the species level. As a result, the reports might include bites from other mosquito species or insects, not necessarily from *Cx. pipiens* mosquitoes. Additionally, there are multiple species complexes of *Cx. pipiens* mosquitoes, such as *Cx. pipiens* and *Cx. pipiens molestus*, which can only be identified through molecular techniques [3]. For now, MA does not differentiate *Cx. pipiens* mosquitoes by complexes. While this study has some of these limitations, the knowledge gained and the rapid and massive data acquisition clearly show the potential and clear utility of citizen science data for mosquito surveillance.

The surge in MA reports following the press release underscores the value of citizen science in monitoring mosquito activity. The high level of public engagement during the 1-month period did provide a comprehensive dataset for creating predictive models, enabling a nuanced understanding of spatial mosquito dynamics across the Netherlands. This approach highlights the potential for leveraging citizen contributions to enhance surveillance efforts and inform targeted intervention strategies. In regions where citizen science data has been collected for many years, one can use the bite reports to investigate biting anomalies that could target interventions. More temporal data would enrich these models even more, so increased and sustained marketing efforts throughout the year would be necessary. In addition, collecting more data might aid in creating minimum data requirement protocols (MRPs) to minimize the impact of convenience sampling biases in citizen science-based modeling [98, 99]. For instance, establishing minimum and maximum reporting thresholds per area and sampling intervals can help create better predictive maps by minimizing self-selection biases from hyper-motivated participants or successful engagement campaigns, or large under-sampling in some areas that would limit data representativeness.

Future research to incorporate citizen science data into current vector models using traditional sampling methods would be beneficial in creating more robust predictions of mosquito vectors in the Netherlands [100]. However, it should be noted that adult *Cx. pipiens* mosquitoes found through MA are not trapped in the traditional way, as most studies do to assess *Cx. pipiens* distribution and abundance [101–103]. Instead of a burden, however, this might represent an opportunity. A previous citizen science project in Germany has shown that most *Culex* mosquito reports are submitted from people’s homes, while traditional traps are placed outdoors and not directly around residents’ homes [77]. In this way, data from citizen scientists might represent a better inference of mosquito exposure rather than mosquito abundance, highlighting the need for more studies to examine where people are more likely to be exposed to mosquitoes. These combined data collections in models could help better identify the factors influencing human–mosquito interactions and bite frequency, which may not necessarily be the same as those modulating mosquito abundance, thus providing valuable insights for targeted intervention and monitoring efforts to manage mosquito populations and mitigate mosquito biting activity.

Additionally, including more human behavioral components in the models may better explain mosquito exposure and biting activity [104]. For instance, using long-term information related to human perception of mosquitoes and MBDs by using the MosquitoWise survey to collect these data might increase the explained variance for citizen science-based models, especially when it comes to mosquitoes biting humans [97, 105]. In addition, incorporating epidemiological data on MBDs (such as WNV and USUV) from birds, humans, and other host populations into these citizen science models and maps can be a further step to identify potential hotspots for disease spread and to enhance early warning systems. Rather than replacing traditional surveillance, these reports could complement existing monitoring programs and aid in public health decision-making by identifying areas of heightened concern where further sampling or vector control measures should be prioritized.

Naturally, there can be some challenges when combining citizen science data with traditional data. Citizen science data might be sporadic, depending on participant availability and interest (i.e., convenience sampling), whereas traditional data often follows a consistent temporal sampling schedule. This disparity can create gaps or over-representation of certain periods. Traditional data collection sites are often predefined and may cover specific landscapes (e.g., urban, peri-urban, rural). In contrast, citizen science data is typically clustered around populated areas, but also can cover gaps in less populated or inaccessible regions. This is where establishing MRPs and other bias-correction methods (scale-dependent sampling effort models) can aid in overcoming these challenges and standardizing the citizen science data so that it can be more easily combined with traditional data to leverage integrated predictive models. The union of traditional data collection, citizen science data, and several factors (such as epidemiological, ecological, and sociological variables) in these models would greatly contribute to our understanding of mosquito–human dynamics and prevention of MBD outbreaks.

## Conclusions

Using citizen science data, this study reveals that the eastern regions of the Netherlands have high mosquito estimates whereas densely populated urban areas and national parks have lower estimates in August. These results were similar to those found by studies using more traditional trapping methods. Bite activity appeared more generally spread throughout the country for the same period, but with more nuanced variability on a local scale. By highlighting sociodemographic factors such as population density and income, as well as environmental variables such as agricultural areas, our findings provide insights into drivers of mosquito abundance and activity. While this study only provides a spatial snapshot of the situation of *Cx. pipiens* during summer in the Netherlands, it is a springboard for innovation in mosquito surveillance modeling. Citizen science data can complement traditional surveillance methods to create more robust and real-time predictive models by filling knowledge gaps and overcoming challenges associated with traditional data collection.

## Supplementary Information

Additional file 1. Supplemental model analysis and results as described in manuscript methods.

## Data Availability

No datasets were generated or analyzed during the current study.

## Abbreviations

MBDs: Mosquito-borne diseases
WNV: West Nile virus
USUV: Usutu virus
MA: Mosquito Alert
GLMMs: Generalized linear mixed models
NOS: Nederlandse Omroep Stichting (a Dutch national news channel)
VIF: Variance inflation factor
AIC: Akaike information criterion
BIC: Bayesian information criterion
Pseudo-R^2^: Pseudo R-squared (a statistical measure of model fit)
UHIs: Urban heat islands
MRPs: Minimum data requirement protocols

## References

[1] Martinet JP, Ferté H, Failloux AB, Schaffner F, Depaquit J. Mosquitoes of North-Western Europe as potential vectors of arboviruses: a review. Viruses. 2019;11:1059. 31739553 10.3390/v11111059 PMC6893686

[2] Fros JJ, Miesen P, Vogels CB, Gaibani P, Sambri V, Martina BE, et al. Comparative Usutu and West Nile virus transmission potential by local *Culex pipiens* mosquitoes in north-western Europe. One Health. 2015;6:31–6. 10.1016/j.onehlt.2015.08.002 PMC5441354 28616462

[3] Vogels CBF, Fros JJ, Göertz GP, Pijlman GP, Koenraadt CJM. Vector competence of northern European *Culex pipiens* biotypes and hybrids for West Nile virus is differentially affected by temperature. Parasit Vectors. 2016;9:393. 27388451 10.1186/s13071-016-1677-0 PMC4937539

[4] Camp JV, Kolodziejek J, Nowotny N. Targeted surveillance reveals native and invasive mosquito species infected with Usutu virus. Parasit Vectors. 2019;12:46. 30665453 10.1186/s13071-019-3316-z PMC6341546

[5] Bakonyi T, Haussig JM. West Nile virus keeps on moving up in Europe. Eurosurveillance. 2020;25:2001938. 33213684 10.2807/1560-7917.ES.2020.25.46.2001938 PMC7678036

[6] Rijks J, Kik M, Slaterus R, Foppen R, Stroo A, IJzer J, et al. Widespread Usutu virus outbreak in birds in the Netherlands, 2016. Euro Surveill. 2016;21:30391. 27918257 10.2807/1560-7917.ES.2016.21.45.30391 PMC5144937

[7] Oude Munnink BB, Münger E, Nieuwenhuijse DF, Kohl R, Van Der Linden A, Schapendonk CME, et al. Genomic monitoring to understand the emergence and spread of Usutu virus in the Netherlands, 2016–2018. Sci Rep. 2020;10:2798. 32071379 10.1038/s41598-020-59692-y PMC7029044

[8] Sikkema RS, Schrama M, van den Berg T, Morren J, Munger E, Krol L, et al. Detection of West Nile virus in a common whitethroat (*Curruca communis*) and *Culex* mosquitoes in the Netherlands, 2020. Euro Surveill. 2020;25:2001704. 33034280 10.2807/1560-7917.ES.2020.25.40.2001704 PMC7545818

[9] Vlaskamp DR, Thijsen SF, Reimerink J, Hilkens P, Bouvy WH, Bantjes SE, et al. First autochthonous human West Nile virus infections in the Netherlands, July to August 2020. Eurosurveillance. 2020;25:2001904. 33213687 10.2807/1560-7917.ES.2020.25.46.2001904 PMC7678035

[10] *Culex pipiens*—factsheet for experts. 2020. https://www.ecdc.europa.eu/en/infectious-disease-topics/related-public-health-topics/disease-vectors/facts/mosquito-factsheets/culex-pipiens. Accessed 10 Aug 2024.

[11] Ibañez-Justicia A, Stroo A, Dik M, Beeuwkes J, Scholte EJ. National mosquito (Diptera: Culicidae) Survey in The Netherlands 2010–2013. J Med Entomol. 2015;52:185–98. 26336303 10.1093/jme/tju058

[12] Rakotoarinia MR, Blanchet FG, Gravel D, Lapen DR, Leighton PA, Ogden NH, et al. Effects of land use and weather on the presence and abundance of mosquito-borne disease vectors in a urban and agricultural landscape in Eastern Ontario, Canada. PLoS ONE. 2022;17:e0262376. 35271575 10.1371/journal.pone.0262376 PMC8912203

[13] Ferraguti M, Martínez-de la Puente J, Roiz D, Ruiz S, Soriguer R, Figuerola J. Effects of landscape anthropization on mosquito community composition and abundance. Sci Rep. 2016;6:29002. 27373794 10.1038/srep29002 PMC4931447

[14] Hunt SK, Galatowitsch ML, McIntosh AR. Interactive effects of land use, temperature, and predators determine native and invasive mosquito distributions. Freshw Biol. 2017;62:1564–77.

[15] Ramasamy R, Surendran SN. Mosquito vectors developing in atypical anthropogenic habitats: global overview of recent observations, mechanisms and impact on disease transmission. J Vector Borne Dis. 2016;53:91. 27353577

[16] Schrama M, Hunting ER, Beechler BR, Guarido MM, Govender D, Nijland W, et al. Human practices promote presence and abundance of disease-transmitting mosquito species. Sci Rep. 2020;10:13543. 32782318 10.1038/s41598-020-69858-3 PMC7421943

[17] Steiger DM, Johnson P, Hilbert DW, Ritchie S, Jones D, Laurance SGW. Effects of landscape disturbance on mosquito community composition in tropical Australia. J Vector Ecol. 2012;37:69–76. 22548538 10.1111/j.1948-7134.2012.00201.x

[18] Washburn JO. Regulatory factors affecting larval mosquito populations in container and pool habitats: implications for biological control. J Am Mosq Control Assoc. 1995;11:279–83. 7595462

[19] Jian Y, Silvestri S, Belluco E, Saltarin A, Chillemi G, Marani M. Environmental forcing and density-dependent controls of *Culex pipiens* abundance in a temperate climate (Northeastern Italy). Ecol Model. 2014;24:301–10.

[20] Krol L, Blom R, Dellar M, van der Beek JG, Stroo ACJ, van Bodegom PM, et al. Interactive effects of climate, land use and soil type on *Culex pipiens/torrentium* abundance. One Health. 2023;21:100589. 10.1016/j.onehlt.2023.100589 PMC10320611 37415720

[21] Vinogradova EB. *Culex pipiens pipiens* mosquitoes: taxonomy, distribution, ecology, physiology, genetic, applied importance and control. Sofia: Pensoft Publishers; 2000.

[22] Becker N, Jöst A, Weitzel T. The *Culex pipiens* complex in Europe. J Am Mosq Control Assoc. 2012;28:53–67. 23401944 10.2987/8756-971X-28.4s.53

[23] Vogels CB, Göertz GP, Pijlman GP, Koenraadt CJ. Vector competence of European mosquitoes for West Nile virus. Emerg Microbes Infect. 2017;6:1–13. 10.1038/emi.2017.82 PMC5717085 29116220

[24] Vilibic-Cavlek T, Savic V, Petrovic T, Toplak I, Barbic L, Petric D, et al. Emerging trends in the epidemiology of West Nile and Usutu virus infections in Southern Europe. Front Vet Sci. 2019;6:437. 31867347 10.3389/fvets.2019.00437 PMC6908483

[25] Nikolay B. A review of West Nile and Usutu virus co-circulation in Europe: how much do transmission cycles overlap? Trans R Soc Trop Med Hyg. 2015;109:609–18. 26286946 10.1093/trstmh/trv066

[26] Pepin KM, Marques-Toledo C, Scherer L, Morais MM, Ellis B, Eiras AE. Cost-effectiveness of novel system of mosquito surveillance and control, Brazil. Emerg Infect Dis. 2013;19:542–50. 23628282 10.3201/eid1904.120117 PMC3647717

[27] Vazquez-Prokopec GM, Chaves LF, Ritchie SA, Davis J, Kitron U. Unforeseen costs of cutting mosquito surveillance budgets. PLoS Negl Trop Dis. 2010;4:e858. 21049010 10.1371/journal.pntd.0000858 PMC2964299

[28] Kampen H, Medlock JM, Vaux AG, Koenraadt CJ, Van Vliet AJ, Bartumeus F, et al. Approaches to passive mosquito surveillance in the EU. Parasit Vectors. 2015;8:1–13. 25567671 10.1186/s13071-014-0604-5 PMC4302443

[29] Palmer JR, Oltra A, Collantes F, Delgado JA, Lucientes J, Delacour S, et al. Citizen science provides a reliable and scalable tool to track disease-carrying mosquitoes. Nat Commun. 2017;8:916. 29066710 10.1038/s41467-017-00914-9 PMC5655677

[30] Bonney R, Cooper CB, Dickinson J, Kelling S, Phillips T, Rosenberg KV, et al. Citizen science: a developing tool for expanding science knowledge and scientific literacy. Bioscience. 2009;59:977–84.

[31] Dickinson JL, Shirk J, Bonter D, Bonney R, Crain RL, Martin J, et al. The current state of citizen science as a tool for ecological research and public engagement. Front Ecol Environ. 2012;10:291–7.

[32] Haywood BK. A “Sense of Place” in public participation in scientific research. Sci Educ. 2014;98:64–83.

[33] Den Broeder L, Devilee J, Van Oers H, Schuit AJ, Wagemakers A. Citizen science for public health. Health Promot Int. 2018;33:505–14. 28011657 10.1093/heapro/daw086 PMC6005099

[34] Sousa LB, Craig A, Chitkara U, Fricker S, Webb C, Williams C, et al. Methodological diversity in citizen science mosquito surveillance: a scoping review. Citiz Sci Theory Pract. 2022;7:8.

[35] Bartumeus F, Oltra A, Palmer JR. Citizen science: a gateway for innovation in disease-carrying mosquito management? Trends Parasitol. 2018;34:727–9. 29793805 10.1016/j.pt.2018.04.010

[36] Eritja R, Ruiz-Arrondo I, Delacour-Estrella S, Schaffner F, Álvarez-Chachero J, Bengoa M, et al. First detection of *Aedes japonicus* in Spain: an unexpected finding triggered by citizen science. Parasit Vectors. 2019;12:1–9. 30674335 10.1186/s13071-019-3317-y PMC6344982

[37] Martínez-Barciela Y, Polina González A, Pereira Martínez JM, Cobo Gradín F, Garrido González J, Abalo Costa X, et al. First record of *Aedes albopictus* in Galicia, obtained by citizen science through Mosquito Alert. Gac Sanit. 2024;S0213–9111:00021–9. 10.1016/j.gaceta.2024.102374 38519323

[38] Južnič-Zonta Ž, Sanpera-Calbet I, Eritja R, Palmer JRB, Escobar A, Garriga J, et al. Mosquito alert: leveraging citizen science to create a GBIF mosquito occurrence dataset. GigaByte. 2022;30:gigabyte54. 10.46471/gigabyte.54 PMC9930537 36824520

[39] Schönenberger AC, Wagner S, Tuten HC, Schaffner F, Torgerson P, Furrer S, et al. Host preferences in host-seeking and blood-fed mosquitoes in Switzerland. Med Vet Entomol. 2016;30:39–52. 26685926 10.1111/mve.12155

[40] Balenghien T, Fouque F, Sabatier P, Bicout DJ. Horse-, bird-, and human-seeking behavior and seasonal abundance of mosquitoes in a West Nile virus focus of southern France. J Med Entomol. 2006;43:936–46. 17017231 10.1603/0022-2585(2006)43[936:hbahba]2.0.co;2

[41] Rizzoli A, Bolzoni L, Chadwick EA, Capelli G, Montarsi F, Grisenti M, et al. Understanding West Nile virus ecology in Europe: *Culex pipiens* host feeding preference in a hotspot of virus emergence. Parasit Vectors. 2015;8:213. 25888754 10.1186/s13071-015-0831-4 PMC4411713

[42] Virgillito C, Longo E, De Marco CM, Serini P, Zucchelli MV, Montarsi F, et al. Involving citizen scientists in monitoring arthropod vectors of human and zoonotic diseases: the case of Mosquito Alert in Italy. Sci Total Environ. 2024;20:174847. 10.1016/j.scitotenv.2024.174847 39025142

[43] MosquitoAlertBCN. https://labs.mosquitoalert.com/MosquitoAlertBCN/. Accessed 10 Aug 2024.

[44] Krol L, Remmerswaal L, Groen M, van der Beek JG, Sikkema RS, Dellar M, et al. Landscape level associations between birds, mosquitoes and microclimates: possible consequences for disease transmission? Parasit Vectors. 2024;26:156. 10.1186/s13071-024-06239-z PMC10964671 38532512

[45] Krol L, Langezaal M, Budidarma L, Wassenaar D, Didaskalou EA, Trimbos K, et al. Distribution of *Culex pipiens* life stages across urban green and grey spaces in Leiden, The Netherlands. Parasit Vectors. 2024;29:37. 10.1186/s13071-024-06120-z PMC10826093 38287368

[46] NOS News. Foto van platgeslagen mug moet onderzoekers helpen: “niet te hard slaan”. 2021. https://nos.nl/artikel/2390287-foto-van-platgeslagen-mug-moet-onderzoekers-helpen-niet-te-hard-slaan. Accessed 10 July 2024.

[47] Barends S, Renes J, Stol T. Het Nederlandse landschap : een historisch-geografische benadering. Matrijs; 1986. https://research.wur.nl/en/publications/het-nederlandse-landschap-een-historisch-geografische-benadering. Accessed 10 Aug 2024.

[48] Ibáñez-Justicia A, Alcaraz-Hernández JD, van Lammeren R, Koenraadt CJM, Bergsma A, Delucchi L, et al. Habitat suitability modelling to assess the introductions of *Aedes albopictus* (Diptera: Culicidae) in the Netherlands. Parasit Vectors. 2020;26:217. 10.1186/s13071-020-04077-3 PMC7184689 32336286

[49] Carrieri M, Fariselli P, Maccagnani B, Angelini P, Calzolari M, Bellini R. Weather factors influencing the population dynamics of *Culex pipiens* (Diptera: Culicidae) in the Po Plain Valley, Italy (1997–2011). Environ Entomol. 2014;43:482–90. 24763101 10.1603/EN13173

[50] Boerlijst S, Johnston E, Ummels A, Krol L, Boelee E, van Bodegom P, et al. Biting the hand that feeds: anthropogenic drivers interactively make mosquitoes thrive. Sci Total Environ. 2023;858:159716. 36302419 10.1016/j.scitotenv.2022.159716

[51] Yang S, Wang LL, Stathopoulos T, Marey AM. Urban microclimate and its impact on built environment—a review. Build Environ. 2023;238:110334.

[52] Statistics Netherlands. Agriculture. CBS Open data StatLine. 2024. https://opendata.cbs.nl/statline/portal.html?_la=en&_catalog=CBS&tableId=80783eng&_theme=1166. Accessed 22 Jan 2025.

[53] Panagos P, Van Liedekerke M, Borrelli P, Köninger J, Ballabio C, Orgiazzi A, et al. European Soil Data Centre 2.0: soil data and knowledge in support of the EU policies. Eur J Soil Sci. 2022;73:e13315.

[54] European Digital Elevation Model (EU-DEM). https://www.eea.europa.eu/en/datahub/datahubitem-view/d08852bc-7b5f-4835-a776-08362e2fbf4b. Accessed 22 Jan 2025.

[55] Centraal Bureau voor de Statistiek. Regionale statistieken. Centraal Bureau voor de Statistiek. https://www.cbs.nl/nl-nl/dossier/nederland-regionaal/regionale-statistieken. Accessed 22 Jan 2025.

[56] Fraction of aquatic species potentially affected by pesticides. European Environment Agency. https://www.eea.europa.eu/data-and-maps/figures/multi-substance-potentially-affected-fraction. Accessed 22 Jan 2025.

[57] Ballabio C, Panagos P, Monatanarella L. Mapping topsoil physical properties at European scale using the LUCAS database. Geoderma. 2016;1:110–23. 10.1016/j.geoderma.2019.113912 PMC6743211 31798185

[58] Panagos P, Van Liedekerke M, Jones A, Montanarella L. European soil data centre: response to European policy support and public data requirements. Land Use Policy. 2012;29:329–38.

[59] Copernicus. Imperviousness Density 2018 (raster 10 m and 100 m), Europe, 3-yearly. https://land.copernicus.eu/en/products/high-resolution-layer-imperviousness/imperviousness-density-2018. Accessed 22 Jan 2025.

[60] Copernicus. Grassland 2018 (raster 10 m and 100 m), Europe, 3-yearly. https://land.copernicus.eu/en/products/high-resolution-layer-grassland/grassland-2018. Accessed 22 Jan 2025.

[61] Copernicus. Water and Wetness status 2018 (raster 10 m and 100 m), Europe, 3-yearly. https://land.copernicus.eu/en/products/high-resolution-layer-water-and-wetness/water-and-wetness-status-2018. Accessed 22 Jan 2025.

[62] Gallego FJ. A population density grid of the European Union. Popul Environ. 2010;31:460–73.

[63] NHI Geodata Portal-Dutch Hydrological Instrumentarium. 2020. https://data.nhi.nu/. Accessed 22 Jan 2025.

[64] Panagos P. The European soil database. GEO Connex. 2006;5:32–3.

[65] Van Liedekerke M, Jones A, Panagos P. ESDBv2 raster library—a set of rasters derived from the European Soil Database Distribution v2. 0. European Commission and the European Soil Bureau Network, CDROM, EUR. 2006;19945.

[66] Copenicus. Water and Wetness status 2018 (raster 10 m and 100 m), Europe, 3-yearly. https://land.copernicus.eu/en/products/high-resolution-layer-water-and-wetness/water-and-wetness-status-2018. Accessed 22 Jan 2025.

[67] Copernicus. Tree Cover Density 2018 (raster 10 m and 100 m), Europe, 3-yearly. https://land.copernicus.eu/en/products/high-resolution-layer-tree-cover-density/tree-cover-density-2018. Accessed 22 Jan 2025.

[68] Dellar M, Sierdsema H, van Bodegom PM, Schrama MJ, Geerling G. The future abundance of key bird species for pathogen transmission in the Netherlands. bioRxiv. 2024. pp. 2024–10. 10.1007/s10393-025-01727-9 PMC12476400 40615625

[69] Royal Netherlands Meteorological Institute. Precipitation—gridded daily precipitation sum in the Netherlands. KNMI Data Platform. https://dataplatform.knmi.nl/dataset/rd1-5. Accessed 22 Jan 2025.

[70] Royal Netherlands Meteorological Institute. Temperature—gridded daily mean temperature in the Netherlands. KNMI Data Platform. https://dataplatform.knmi.nl/dataset/tg1-5. Accessed 22 Jan 2025.

[71] Zuur AF, Ieno EN, Walker NJ, Saveliev AA, Smith GM. Mixed effects models and extensions in ecology with R, vol. 574. Berlin: Springer; 2009.

[72] Anderson D, Burnham K. Model selection and multi-model inference. Vol. 63. Second NY: Springer-Verlag. 2004. p. 10.

[73] R Core Team. R: The R Project for Statistical Computing. Vienna, Austria; 2021. https://www.r-project.org/. Accessed 22 Jan 2025.

[74] Obenauer JF, Andrew Joyner T, Harris JB. The importance of human population characteristics in modeling *Aedes aegypti* distributions and assessing risk of mosquito-borne infectious diseases. Trop Med Health. 2017;15:38. 10.1186/s41182-017-0078-1 PMC5688614 29167627

[75] Gubler DJ. Dengue, urbanization and globalization: the unholy trinity of the 21st century. Trop Med Health. 2011;39:S3-11. 10.2149/tmh.2011-S05 PMC3317603 22500131

[76] Arsenault-Benoit A, Fritz ML. Spatiotemporal organization of cryptic North American *Culex* species along an urbanization gradient. Ecol Solut Evid. 2023;4:e12282. 38898889 10.1002/2688-8319.12282 PMC11185319

[77] Pernat N, Kampen H, Jeschke JM, Werner D. Buzzing homes: using citizen science data to explore the effects of urbanization on indoor mosquito communities. Insects. 2021;12:374. 33919337 10.3390/insects12050374 PMC8143366

[78] Zettle M, Anderson E, LaDeau SL. Changes in container-breeding mosquito diversity and abundance along an urbanization gradient are associated with dominance of arboviral vectors. J Med Entomol. 2022;59:843–54. 35388898 10.1093/jme/tjac023

[79] LaDeau SL, Leisnham PT, Biehler D, Bodner D. Higher mosquito production in low-income neighborhoods of Baltimore and Washington, DC: understanding ecological drivers and mosquito-borne disease risk in temperate cities. Int J Environ Res Public Health. 2013;10:1505–26. 23583963 10.3390/ijerph10041505 PMC3709331

[80] Reisen WK, Meyer RP, Tempelis CH, Spoehel JJ. Mosquito abundance and bionomics in residential communities in Orange and Los Angeles Counties, California. J Med Entomol. 1990;27:356–67. 1970608 10.1093/jmedent/27.3.356

[81] Vogels CBF, Möhlmann TWR, Melsen D, Favia G, Wennergren U, Koenraadt CJM. Latitudinal diversity of *Culex pipiens* biotypes and hybrids in farm, peri-urban, and wetland habitats in Europe. PLoS ONE. 2016;11:e0166959. 27870890 10.1371/journal.pone.0166959 PMC5117740

[82] Ferraguti M, Magallanes S, Ibáñez-Justicia A. Implication of human landscape transformation on mosquito populations. In: Gutiérrez-López R, Logan JG, M-d la Puente J, editors. Ecology and control of vector-borne diseases. Wageningen: Wageningen Academic Publishers; 2022. p. 279–83.

[83] Service MW. The taxonomy and biology of two sympatric sibling species of *Culex*, *C. pipiens* and *C. torrentium* (Diptera, Culicidae). J Zool. 1968;156:313–23.

[84] Börstler J, Lühken R, Rudolf M, Steinke S, Melaun C, Becker S, et al. The use of morphometric wing characters to discriminate female *Culex pipiens* and *Culex torrentium*. J Vector Ecol. 2014;39:204–12. 24820574 10.1111/j.1948-7134.2014.12088.x

[85] Blom R, Krol L, Langezaal M, Schrama M, Trimbos KB, Wassenaar D, et al. Blood-feeding patterns of *Culex pipiens* biotype pipiens and pipiens/molestus hybrids in relation to avian community composition in urban habitats. Parasit Vectors. 2024;17:95. 38424573 10.1186/s13071-024-06186-9 PMC10902945

[86] Wehmeyer ML, Jaworski L, Jöst H, Șuleșco T, Rauhöft L, Afonso SMM, et al. Host attraction and host feeding patterns indicate generalist feeding of *Culex pipiens* s.s. and *Cx. torrentium*. Parasit Vectors. 2024;17:369. 39215365 10.1186/s13071-024-06439-7 PMC11363403

[87] Rejmánková E, Grieco J, Achee N, Roberts DR. Ecology of larval habitats. In: Manguin S, editor. Anopheles mosquitoes-new insights into malaria vectors. London: InTech; 2013. p. 397–446.

[88] Arnfield AJ. Two decades of urban climate research: a review of turbulence, exchanges of energy and water, and the urban heat island. Int J Climatol. 2003;23:1–26.

[89] Townroe S, Callaghan A. British container breeding mosquitoes: the impact of urbanisation and climate change on community composition and phenology. PLoS ONE. 2014;9:e95325. 24759617 10.1371/journal.pone.0095325 PMC3997353

[90] Oke TR. City size and the urban heat island. Atmos Environ. 1973;7:769–79.

[91] Hongoh V, Berrang-Ford L, Scott M, Lindsay L. Expanding geographical distribution of the mosquito, *Culex pipiens*, in Canada under climate change. Appl Geogr. 2012;33:53–62.

[92] Barends S, Renes J, Stol T. Het Nederlandse landschap: een historisch-geografische benadering. 10th ed. Matrijs; 2010.

[93] Krol L, Dellar M, Ibáñez-Justicia A, Schrier G van der, Schrama M, Geerling GW, et al. Combined effects of future climate and land use change on mosquitoes: the distribution of *Culex pipiens* under One Health scenarios in the Netherlands. Research Square. 2024. https://www.researchsquare.com/article/rs-5298493/v1. Accessed 22 Jan 2025.

[94] Esser HJ, Liefting Y, Ibáñez-Justicia A, van der Jeugd H, van Turnhout CAM, Stroo A, et al. Spatial risk analysis for the introduction and circulation of six arboviruses in the Netherlands. Parasit Vectors. 2020;13:464. 32912330 10.1186/s13071-020-04339-0 PMC7488554

[95] Wang J, Ogden NH, Zhu H. The Impact of weather conditions on *Culex pipiens* and *Culex restuans* (Diptera: Culicidae) abundance: a case study in Peel Region. J Med Entomol. 2011;48:468–75. 21485391 10.1603/me10117

[96] Kilpatrick AM, Meola MA, Moudy RM, Kramer LD. Temperature, viral genetics, and the transmission of West Nile virus by Culex pipiens mosquitoes. PLoS Pathog. 2008;4:e1000092. 18584026 10.1371/journal.ppat.1000092 PMC2430533

[97] de Best PA, Abourashed A, Doornekamp L, van Gorp ECM, Timen A, Sikkema RS, et al. Determinants of intended prevention behaviour against mosquitoes and mosquito-borne viruses in the Netherlands and Spain using the MosquitoWise survey: cross-sectional study. BMC Public Health. 2024;24:1781. 38965485 10.1186/s12889-024-19293-0 PMC11223381

[98] van Strien AJ, van Swaay CAM, Termaat T. Opportunistic citizen science data of animal species produce reliable estimates of distribution trends if analysed with occupancy models. J Appl Ecol. 2013;50:1450–8.

[99] Henckel L, Bradter U, Jönsson M, Isaac NJB, Snäll T. Assessing the usefulness of citizen science data for habitat suitability modelling: opportunistic reporting versus sampling based on a systematic protocol. Divers Distrib. 2020;26:1276–90.

[100] Robinson OJ, Ruiz-Gutierrez V, Reynolds MD, Golet GH, Strimas-Mackey M, Fink D. Integrating citizen science data with expert surveys increases accuracy and spatial extent of species distribution models. Divers Distrib. 2020;26:976–86.

[101] Ijumba JN, Lindsay SW. Impact of irrigation on malaria in Africa: paddies paradox. Med Vet Entomol. 2001;15:1–11. 11297093 10.1046/j.1365-2915.2001.00279.x

[102] Bowden SE, Magori K, Drake JM. Regional differences in the association between land cover and West Nile virus disease incidence in humans in the United States. Am J Trop Med Hyg. 2011;84:234. 21292890 10.4269/ajtmh.2011.10-0134 PMC3029173

[103] Eisen L, Barker CM, Moore CG, Pape WJ, Winters AM, Cheronis N. Irrigated agriculture is an important risk factor for West Nile virus disease in the hyperendemic Larimer-Boulder-Weld area of north central Colorado. J Med Entomol. 2010;47:939–51. 20939393 10.1603/me10036

[104] Thongsripong P, Hyman JM, Kapan DD, Bennett SN. Human-mosquito contact: a missing link in our understanding of mosquito-borne disease transmission dynamics. Ann Entomol Soc Am. 2021;114:397–414. 34249219 10.1093/aesa/saab011 PMC8266639

[105] Abourashed A, de Best PA, Doornekamp L, Sikkema RS, van Gorp ECM, Timen A, et al. Development and validation of the MosquitoWise survey to assess perceptions towards mosquitoes and mosquito-borne viruses in Europe. Sci Rep. 2024;14:1777. 38245571 10.1038/s41598-024-52219-9 PMC10799950

[106] Mosquito Alert. Mosquito alert user agreement. 2017. https://www.mosquitoalert.com/en/user-agreement/. Accessed 22 Jan 2025.

