# Supplementary material for: Spatial inference of Culex pipiens abundance and biting activity distribution in the Netherlands using citizen science
Source: Parasit Vectors. 2025 Apr 30;18:162. doi: 10.1186/s13071-025-06774-3 (PMC12042346; doi:10.1186/s13071-025-06774-3)
Supplement: Supplementary file 1 — Additional file 1. Supplemental model analysis and results as described in manuscript methods. [file 13071_2025_6774_MOESM1_ESM.pdf]

Supplemental Analysis for Spatial analysis of *Culex pipiens* abundance and biting activity distribution in the Netherlands using citizen science

Table of Contents

Figure S1. Histograms of the number of Mosquito Alert *Culex pipiens* report with and without outliers, for each 1x1 km grid cell. **3**

Figure S2. Distribution goodnes of fit assessement for *Culex* counts, excluding outliers, using Poisson (red), negative inflated binomial (green), and geometric (blue) distributions. **4**

Figure S3. Validation results (QQ plot of residuals and scatterplot of residuals against predicted values) of *Culex* pipiens report counts predictive model without outliers. **5**

Figure S4. Cross-validation of *Culex* reports model (predicted vs. observed values). **6**

Figure S5. Histograms of the number of Mosquito Alert bite report distributions with and without outliers, for each 1x1 km grid cell. **7**

Figure S6. Distribution goodness of fit assessment for bite counts, excluding outliers, using Poisson (red), negative inflated binomial (green), and geometric (blue) distributions. **8**

Figure S7. Validation results (QQ plot of residuals and scatterplot of residuals against predicted values) of bite report count predictive model without outliers. **9**

Figure S8. Cross-validation of bite model (predicted vs. observed values). **10**

1.1 Identification of Outliers in *Culex pipiens* reports

To improve the robustness of our models, we analyzed potential outliers in the response variables of our datasets. Outliers were identified using a percentile-based approach in regard to the data distribution. We removed observations that exceeded the 90th percentile for *Culex* count distribution. At this percentile, the outlier tests for residuals became non-significant. This cutoff value was selected instead of more common choices (e.g. IQRx1.5) to effectively exclude extreme values while preserving the majority of the data (Fig. S1). In any case, we also tested that selecting IQRx1.5, and hence removing many more outliers, only slightly improved the model, and we obtained the same results.

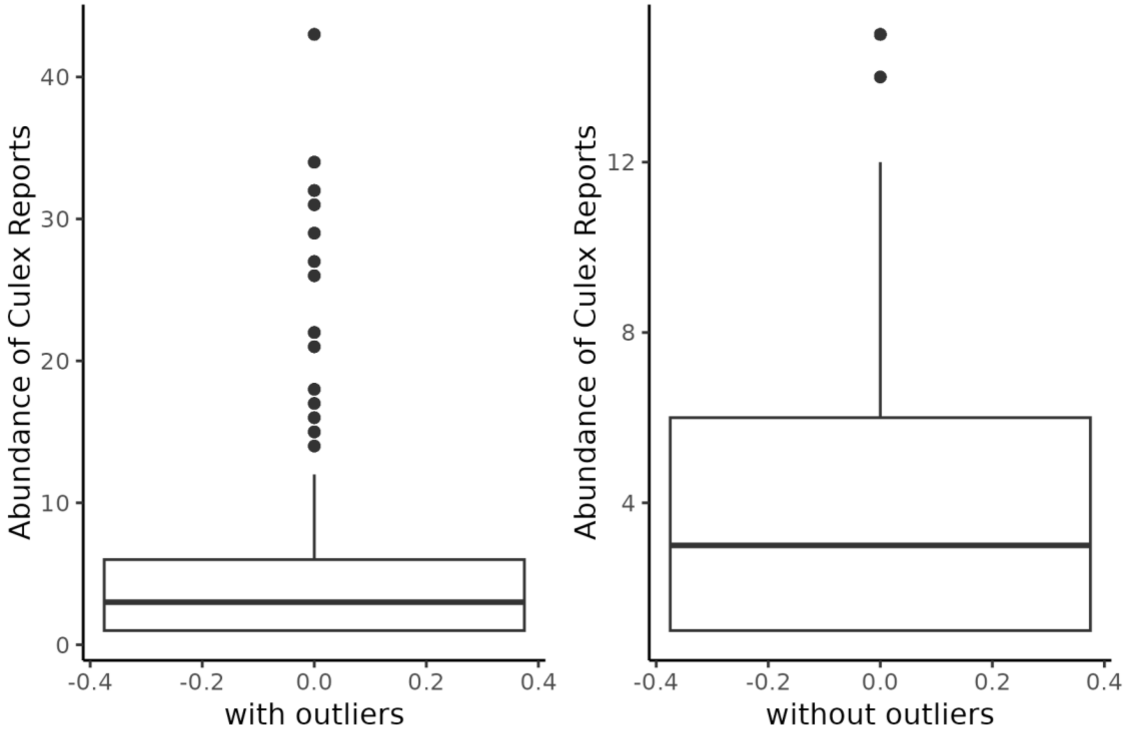

Figure S1. Histograms of the number of Mosquito Alert *Culex pipiens* report with and without outliers, for each 1x1 km grid cell. Outliers were removed based on the 90th percentile of the *Culex* counts distribution.

1.2 Distribution of *Culex pipiens* reporting counts

We explored the initial distribution of the response variables using visual tools, which indicated that the variable displayed over-dispersion—where the variance exceeds the mean—a common characteristic in biological data. To identify the best fitting distribution for our response variable, we evaluated three count-based distributions: Poisson, Negative Binomial, and Geometric. To confirm our selection of the Negative Binomial distribution, we calculated goodness-of-fit metrics, specifically the log-likelihood. The best-fit parameters for each distribution are summarized in Fig. S2.

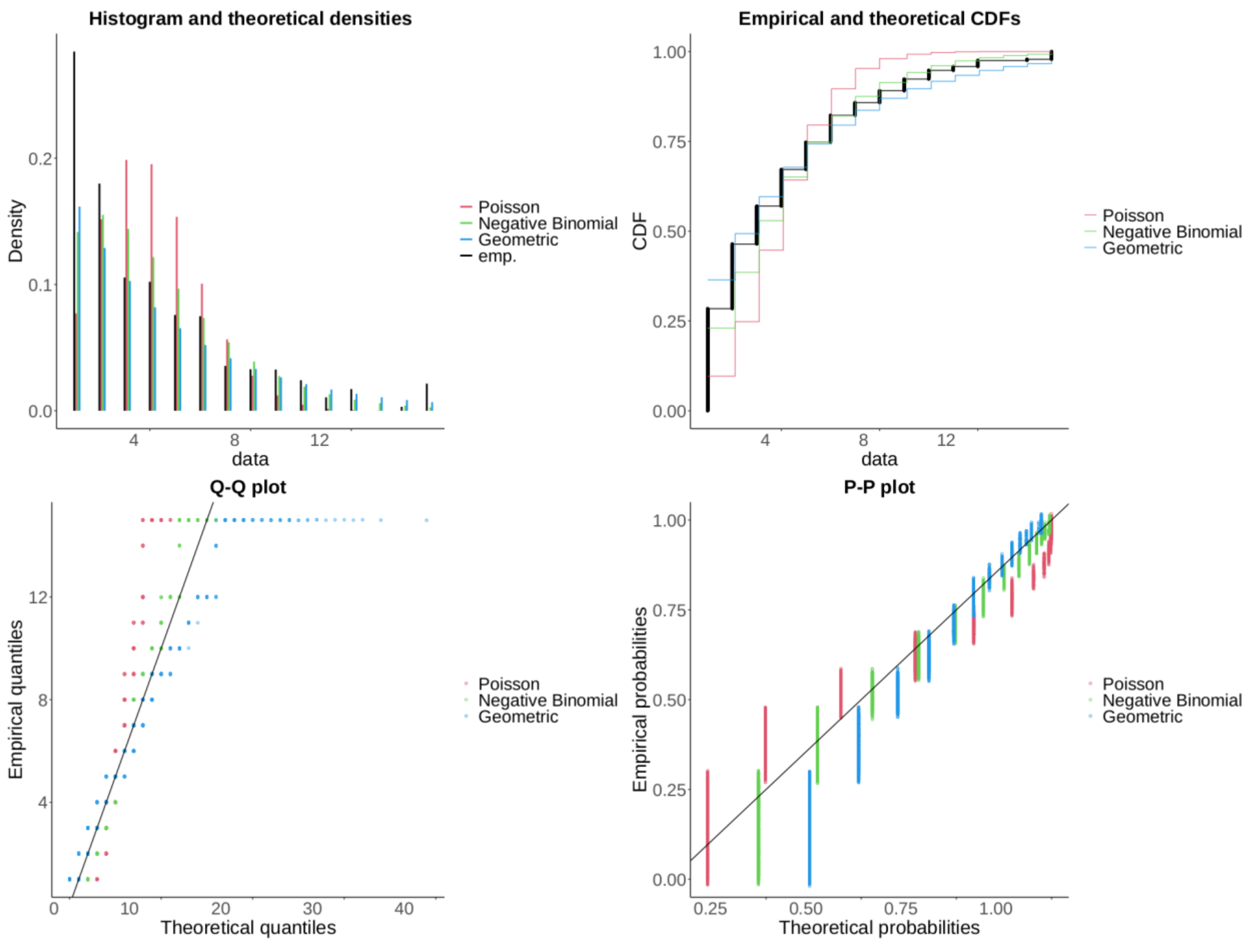

Figure S2. Distribution goodnes of fit assessement for *Culex* counts, excluding outliers, using Poisson (red), negative inflated binomial (green), and geometric (blue) distributions. Based on this assessment, we concluded that the Negative binomial was the best fit model, so we used it as the underlying response data distribution in our GLMMs. Best-fit parameters are as follows: Poisson distribution (λ = 3.93), Negative Binomial distribution (μ = 3.93, θ = 2.69), and Geometric distribution (p = 0.20). Based on the log-likelihood values, the Negative Binomial distribution (LogLik = -9851.457) provides the best fit to the empirical data, outperforming the Poisson distribution (LogLik = -11108.77) and the Geometric distribution (LogLik = -10299.92).

1.3 *Culex* Model Validation

To evaluate the adequacy of the fitted models, we examine the QQ-plots (Fig. S3 left), and residuals vs. predicted plots (Fig. S3 right). The QQ-plot compares the distribution of model residuals to a uniform theoretical distribution because, ideally, the residuals should be uniformly distributed. In our case, the QQ plot showed that residuals did not systematically increase or decrease depending on the observed values. However, residuals were all above the predicted vs. observed one-to-one theoretical line, indicating a slight but consistent underestimation in our predictions of the observed data.

Based on the tests illustrated on QQ-plot, we confirmed that there is neither overdispersion nor underdispersion, and no presence of outliers. Nevertheless, the Kolmogorov-Smirnov test showed a poor goodness of fit, which may be attributed to the large number of observations that we have.

In addition, the Residuals vs. Predicted plot (Fig. S3 right) suggests a systematic error in the models when any patterns are observed. In our case, the residuals were randomly scattered around zero without any clear pattern. However, the plot reveals that the model tends to struggle to predict small values accurately, as residuals become larger. This is so because there is less data in those lower numbers.

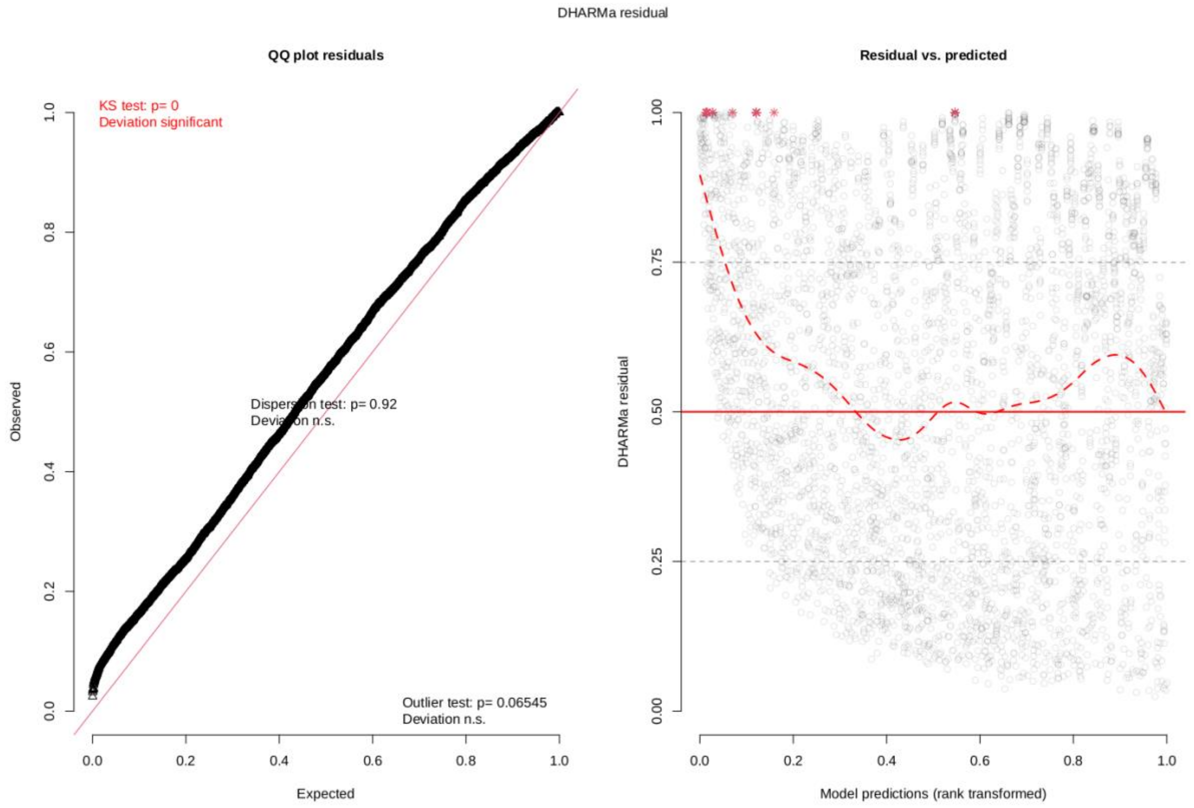

Figure S3. Validation results (QQ plot of residuals and scatterplot of residuals against predicted values) of *Culex* pipiens report counts predictive model without outliers.

Cross-validation was conducted to assess the predictive performance of the Culex model (refer to the Main Text). As illustrated in Fig. S4, there is a strong positive relationship between the predicted and observed values. The slope of the regression line is less than 1, which suggests that the model tends to underestimate higher counts while slightly overestimating lower counts. Additionally, the high Pearson's and Spearman's rank correlations, both around 0.7, indicate that the model captures a significant portion of the explained variation.

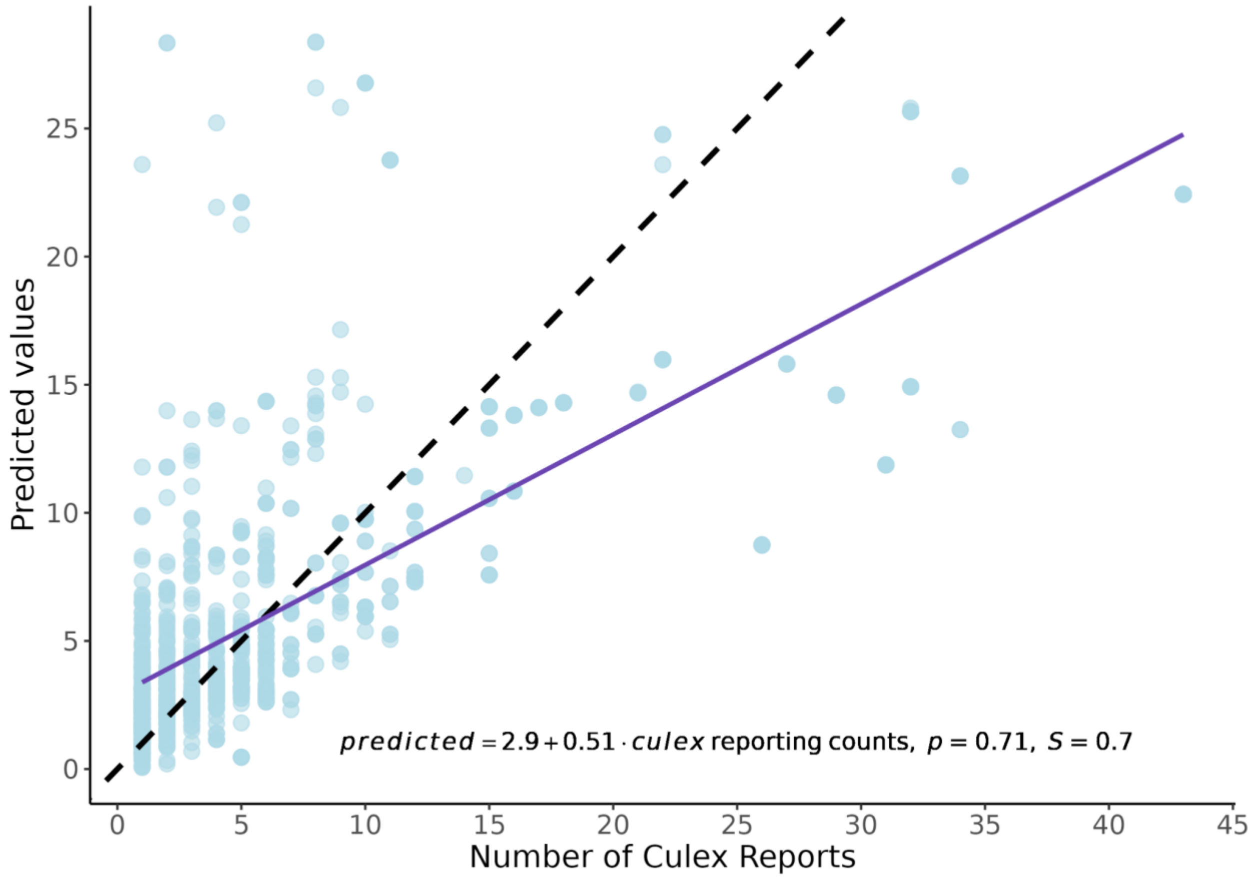

Figure S4. Cross-validation of *Culex* reports model (predicted vs. observed values). Predicted values were obtained from the model trained on 80% of the dataset, while the observed values (*Culex* reports) came from the remaining 20% of the original data. The dashed black line represents the 1:1 line, illustrating the perfect agreement between predictions and observations. Purple line indicates the linear regression between the predicted and observed values.

2.1. Identification of outliers in bite reports

Following a similar procedure detailed above, we removed observations that exceeded the 85th percentile for bite count distribution. At this percentile, the outlier tests for residuals became non-significant. This cutoff value, was selected instead of more common choices (e.g. IQRx1.5) or previous choices as for the *Culex pipiens* (i.e. 90%), to effectively exclude extreme values while preserving the majority of the data (Fig. S1). In any case, we tested that selecting IQRx1.5, and, hence removing many more outliers, did not improve the model and we obtain the same results.

This cutoff value was chosen to effectively eliminate extreme values while keeping most of the data intact (Fig. S5).

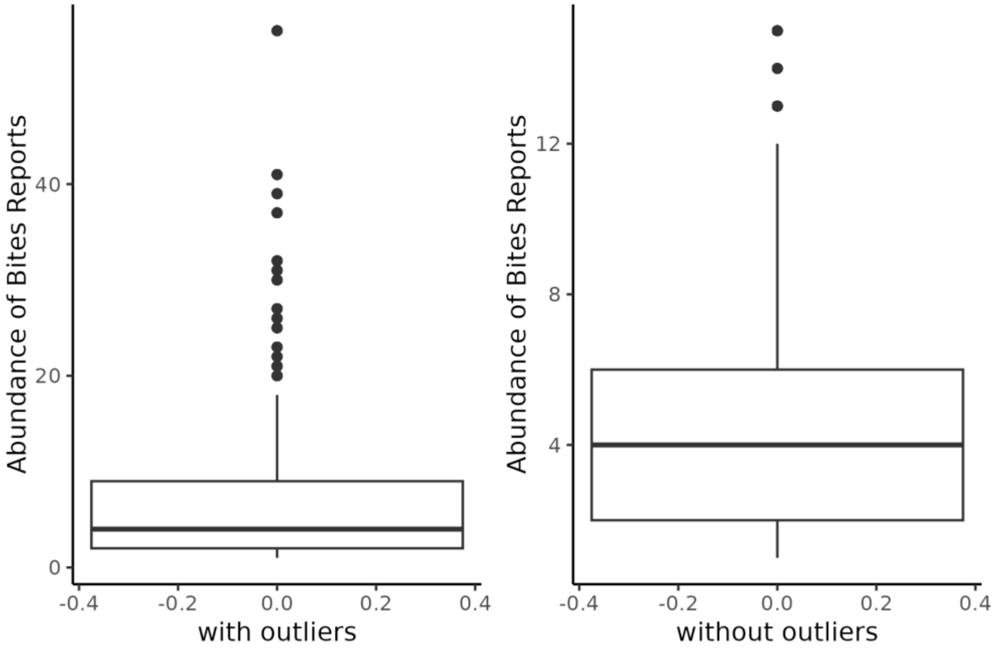

Figure S5. Histograms of the number of Mosquito Alert bite report distributions with and without outliers, for each 1x1 km grid cell. Outliers were removed based on the 85th percentile of the bites count distribution.

2.2 Distribution of bites reporting counts

We evaluated the distribution of bites reporting counts, considering three count-based distributions: Poisson, Negative Binomial, and Geometric. Negative Binomial distribution was selected as the most appropriate for the data (see Fig. S6).

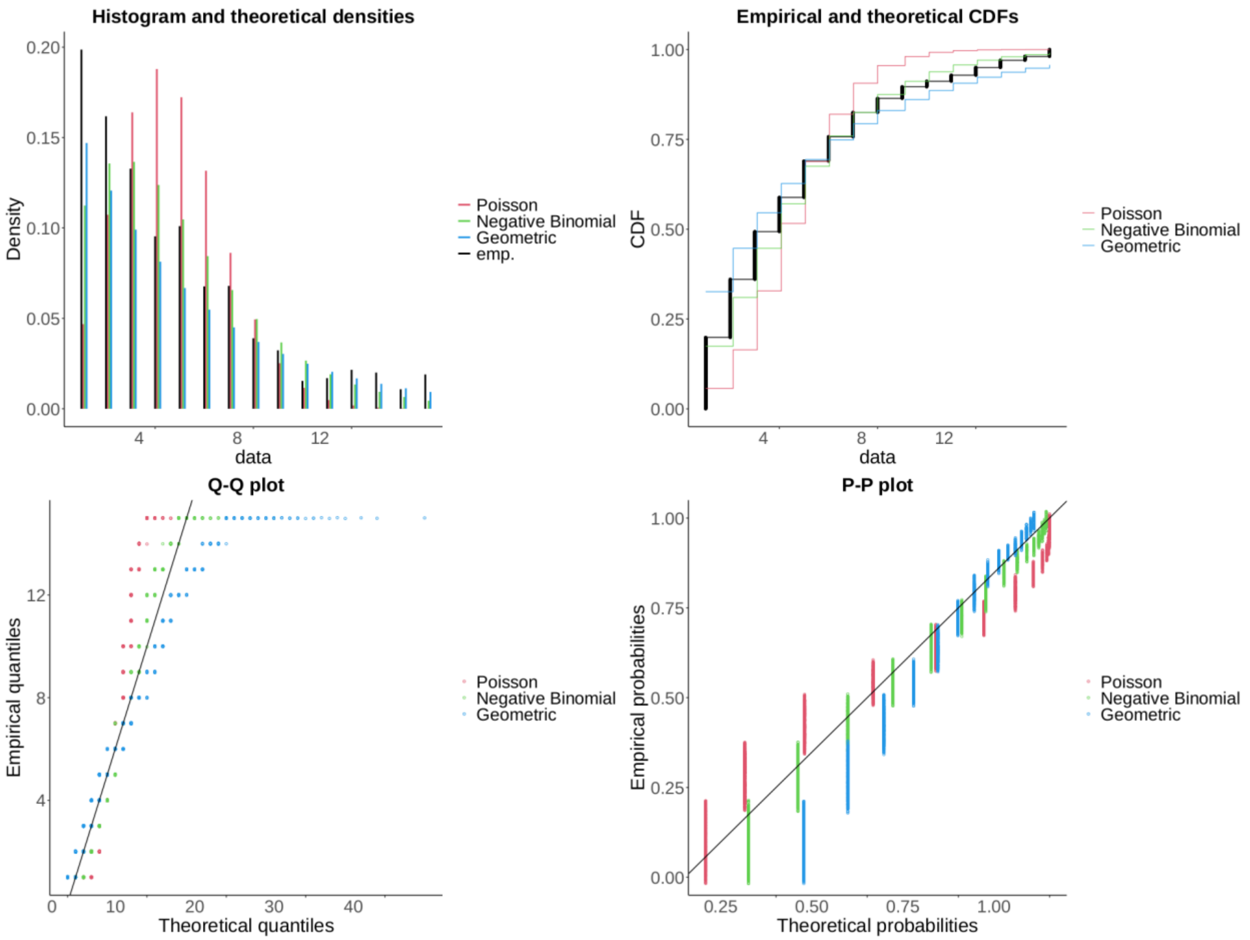

Figure S6. Distribution goodness of fit assessment for bite counts, excluding outliers, using Poisson (red), negative inflated binomial (green), and geometric (blue) distributions. Based on this assessment, we concluded that the Negative binomial was the best fit model, so we used it as the underlying response data distribution in our GLMMs. Best-fit parameters are as follows: Poisson distribution (λ = 4.58), Negative Binomial distribution (μ = 4.58, θ = 2.98), and Geometric distribution (p = 0.18). Based on the log-likelihood values, the Negative Binomial distribution (LogLik = -9717.699) provides the best fit to the empirical data, outperforming the Poisson distribution (LogLik = -10929.53) and the Geometric distribution (LogLik = -10238.46).

2.3 Model validation

The QQ and residuals versus predicted plots for the bites reporting model displayed patterns similar to those observed in the *Culex* reporting model validation (Fig. S7). Although the model demonstrated a poor goodness of fit, no distinct patterns were evident in the residuals other than a slight but consistent underestimation of predicted values. Additionally, this model struggled to accurately predict small values. Cross-validation results indicated a correlation between predicted and observed values that mirrored the findings from the *Culex* reporting model, showing that the predictions closely aligned with the observations in both magnitude and order (Fig. S8).

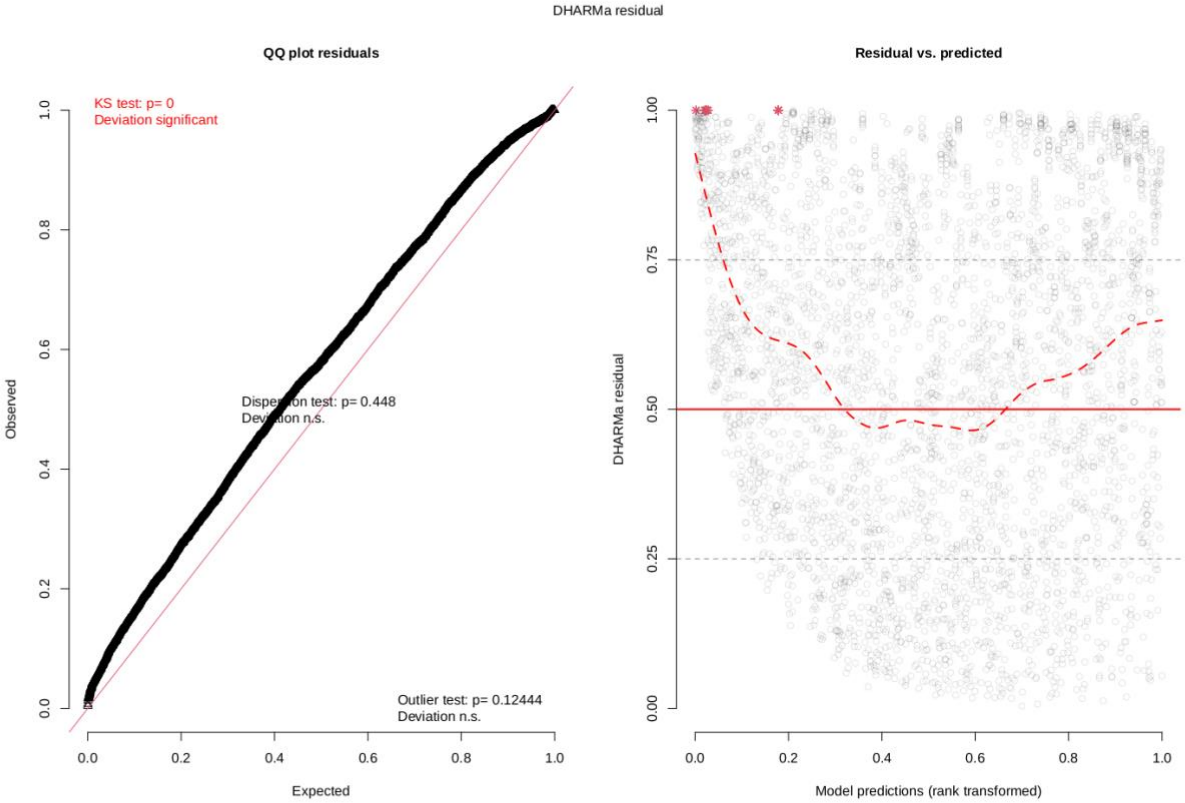

Figure S7. Validation results (QQ plot of residuals and scatterplot of residuals against predicted values) of bite report count predictive model without outliers.

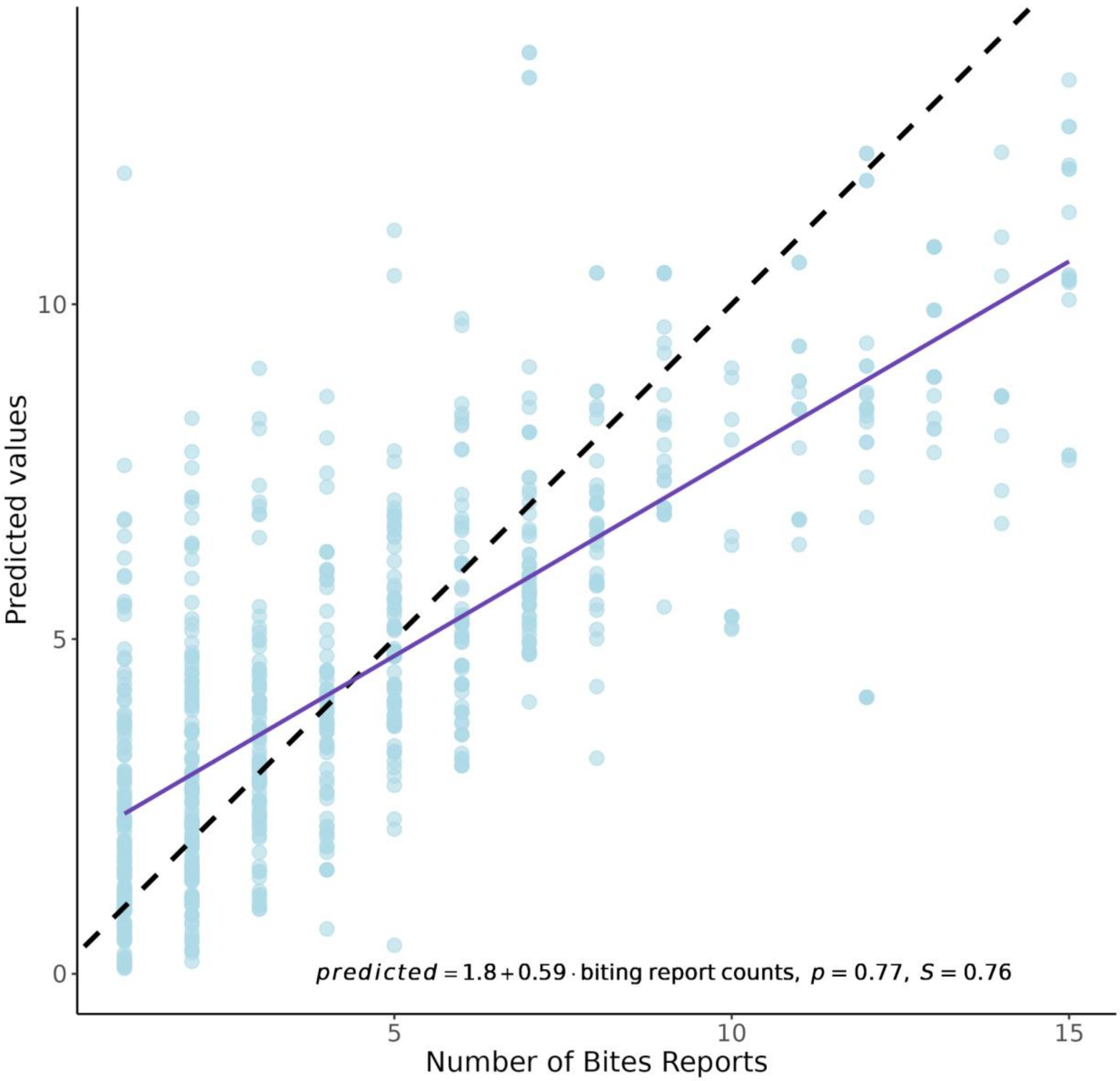

Figure S8. Cross-validation of bite model (predicted vs. observed values). Predicted values were obtained from the model trained on 80% of the dataset, while the observed values (biting report counts) came from the remaining 20% of the original data. The dashed black line represents the 1:1 line, illustrating the perfect agreement between predictions and observations. Purple line indicates the linear regression between the predicted and observed values.
